# Plasma cells in human pancreatic ductal adenocarcinoma secrete antibodies against self-antigens

**DOI:** 10.1172/jci.insight.172449

**Published:** 2023-11-08

**Authors:** Min Yao, Jonathan Preall, Johannes T.-H. Yeh, Darryl Pappin, Paolo Cifani, Yixin Zhao, Sophia Shen, Philip Moresco, Brian He, Hardik Patel, Amber N. Habowski, Daniel A. King, Kara Raphael, Arvind Rishi, Divyesh Sejpal, Matthew J. Weiss, David Tuveson, Douglas T. Fearon

**Affiliations:** 1 Cold Spring Harbor Laboratory and; 2 Cold Spring Harbor High School, Cold Spring Harbor, New York, USA.; 3 Graduate Program in Genetics, Stony Brook University, Stony Brook, New York, USA.; 4 Medical Scientist Training Program, Stony Brook University Renaissance School of Medicine, Stony Brook University, Stony Brook, New York, USA.; 5 North Shore University Hospital, Manhasset, New York, USA.; 6 Weill Cornell Medicine, New York, New York, USA.

**Keywords:** Immunology, Oncology, Adaptive immunity, Antigen, Cancer

## Abstract

Intratumoral B cell responses are associated with more favorable clinical outcomes in human pancreatic ductal adenocarcinoma (PDAC). However, the antigens driving these B cell responses are largely unknown. We sought to discover these antigens by using single-cell RNA sequencing (scRNA-Seq) and immunoglobulin (Ig) sequencing of tumor-infiltrating immune cells from 7 primary PDAC samples. We identified activated T and B cell responses and evidence of germinal center reactions. Ig sequencing identified plasma cell (PC) clones expressing isotype-switched and hypermutated Igs, suggesting the occurrence of T cell–dependent B cell responses. We assessed the reactivity of 41 recombinant antibodies that represented the products of 235 PCs and 12 B cells toward multiple cell lines and PDAC tissues and observed frequent staining of intracellular self-antigens. Three of these antigens were identified: the filamentous actin (F-actin), the nucleic protein RuvB like AAA ATPase 2 (RUVBL2), and the mitochondrial protein heat shock protein family D (Hsp60) member 1 (HSPD1). Antibody titers against F-actin and HSPD1 were substantially elevated in the plasma of patients with PDAC compared with healthy donors. Thus, PCs in PDAC produce autoantibodies reacting with intracellular self-antigens, which may result from promotion of preexisting, autoreactive B cell responses. These observations indicate the chronic inflammatory microenvironment of PDAC can support the adaptive immune response.

## Graphical abstract

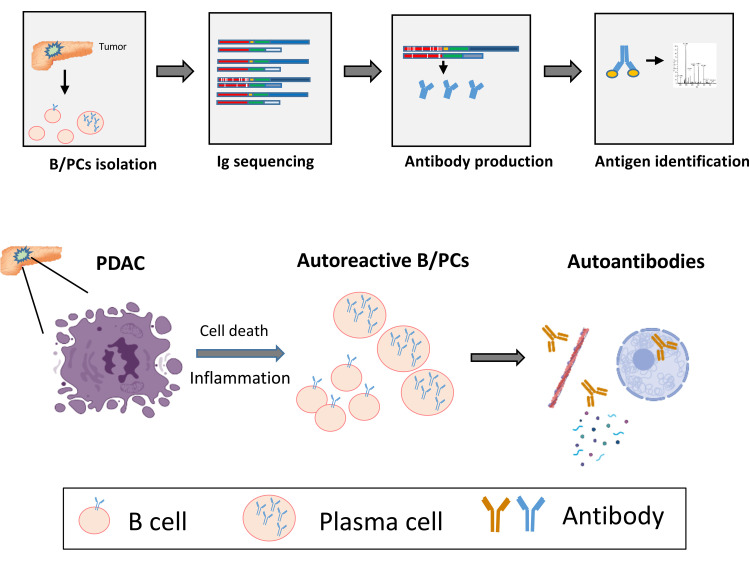

## Introduction

Microsatellite-stable pancreatic ductal adenocarcinoma (PDAC) does not respond to current immunotherapies, has few T cells in cancer cell nests, and has been thought to minimally stimulate the adaptive immune system (1). Recently, however, it has been observed that patients with PDAC whose tumors contained tertiary lymphoid structures (TLSs) with germinal centers, memory B cells, and memory CD4^+^ T cells had improved long-term survival (2–4), suggesting the occurrence of clinically relevant, ongoing anti-PDAC immune responses. The possibility of these immune responses is supported by a study involving patients with PDAC and colorectal cancer in which 1-week continuous administration of an inhibitor of the chemokine receptor CXCR4 revealed ongoing antitumor immune responses (5).

The antigens driving these intratumor immune reactions in human PDAC are unknown. Although PDAC has a low mutational frequency compared with other cancers (1), attention has been directed to T cell clones from PDAC patients with specificity to neoantigens arising from mutations that predicted cross-reactive microbial epitopes (6). The intratumoral immune response may also be directed toward germline-encoded antigens, a concept that has been supported by the finding that immunization of mice with induced pluripotent stem cells confers protection against several tumor models (7, 8). Thus, defining the range of antigens that are driving the intratumoral immune response in PDAC may expand our knowledge of the interaction between this cancer and the immune system.

B cell activation is triggered by antigen interaction with membrane immunoglobulin (Ig). This interaction may lead to B cell activation, antigen presentation to CD4^+^ T cells, and the formation of germinal center reactions. The latter will lead to B cell clonal expansion, heavy (H) chain isotype switching, H chain and light (L) chain variable region somatic hypermutation (SHM), and ultimately differentiation into antibody-secreting plasma cells (PCs). Therefore, antibodies derived from activated B cell responses may help identify those antigens that are driving intratumoral immune responses. In the current report, we have used single-cell RNA sequencing (scRNA-Seq) and Ig sequencing of PCs and B cells from primary PDAC specimens to identify their paired H and L chains, which enabled screening of antibody reactivity.

## Results

### Identification of B and T cell responses by scRNA-Seq in human PDAC.

**Figure 1:**
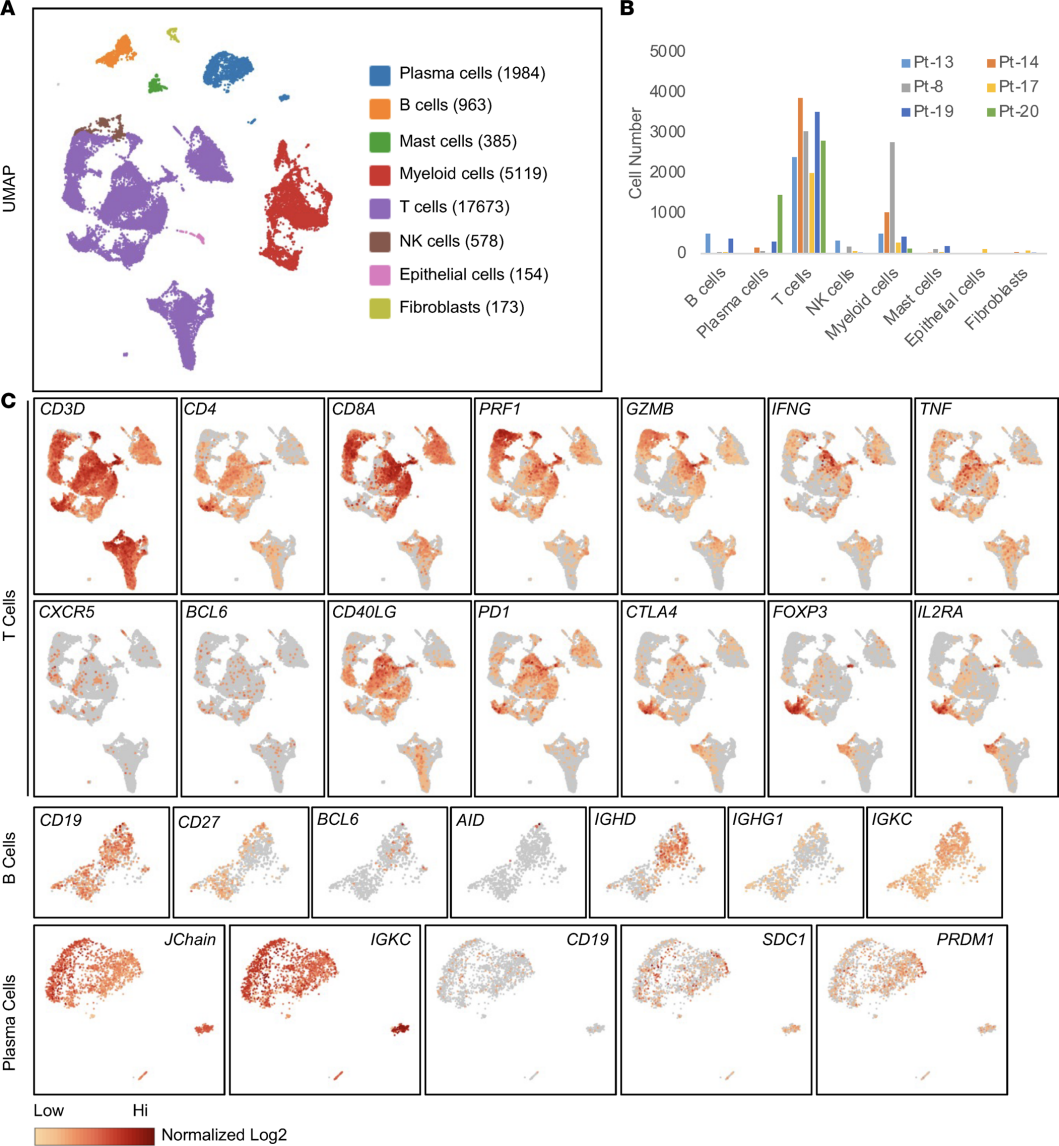
Identification of active T and B cell responses in PDACs by scRNA-Seq. (**A**) UMAP clusters are shown of immune cells isolated from 6 primary PDACs. (**B**) The cell numbers of different immune cell types identified in individual patients are shown. (**C**) The expression of selected genes in T cell, B cell, and PC clusters. The intensity of the colors represents the normalized log_2_ transformation of UMI. UMAP, uniform manifold approximation and projection; UMI, unique molecular identifier.

We performed scRNA-Seq of CD45^+^ immune cells from 7 untreated primary PDAC samples, including 4 microsatellite-stable surgically resected tumors and 3 fine-needle aspiration (FNA) biopsies (Supplemental Table 1; supplemental material available online with this article; https://doi.org/10.1172/jci.insight.172449DS1). Flow cytometry analysis of those 7 samples revealed that the proportion of B cells (CD19^+^CD38^-/lo^) within CD45^+^ immune cells ranged from 1% to 16% and of PCs (CD38^hi^) ranged from 0.4% to 5% (Supplemental Figure 1). scRNA-Seq from 6 samples (scRNA-Seq for patient 15’s [Pt-15] sample technically failed) identified a total of 26,702 immune cells and 327 nonimmune cells. The major immune cells included T cells (66%), myeloid cells (19%), PCs (7.4%), and B cells (3.6%), with T and myeloid cells being the most frequent cell types in each sample (Figure 1, A and B, and Supplemental Figure 2A). The immune cell populations varied among individual samples, as revealed by both flow cytometry and scRNA-Seq (Figure 1B and Supplemental Figure 1B). In the CD8*^+^* T cells, features of T cell activation were prominent, as evidenced by frequent expression of the effector genes *PRF1* (46% of CD8^+^ T cells) and *GZMB* (31%), as well as the cytokines *IFNG* (13%) and *TNF* (11%). Subsets of CD8^+^ T cells also expressed the inhibitory receptors *LAG3* (*27*%), *TIGIT* (22%), *KLRC1* (19%), *PD-1* (14%), and *CTLA4* (3%) (Figure 1C and Supplemental Figure 2B). The expression of these receptors also identifies effector cells. In the CD4*^+^* T cell population, a Th1 response was apparent, as assessed by the expression of the Th1 lineage marker, *TBX21* (13% of CD4^+^ T cells), and the Th1 cytokines, *TNF* (18%) and *IFNG* (6%) (Supplemental Figure 2, C and D). *FOXP3^+^* CD4*^+^* regulatory T cells represented a relatively abundant CD4*^+^* T cell population (22%) and characteristically coexpressed *IL2RA*, *PD-1*, and *CTLA4*. CD4*^+^* T follicular helper T cells (Tfh cells) were identified by their expression of *BCL6* (2%), *IL-21* (1%), and the chemokine receptor *CXCR5* (2%) (Figure 1C and Supplemental Figure 2, C and D). In summary, the PDAC tumors contained activated CD8*^+^* and CD4*^+^* T cells, including Tfh cells of presumed germinal center origin.

The B cell cluster (*n* = 963 cells) was defined by the expression of the pan–B cell markers, *CD19* and *CD20*. We found 35% of B cells expressed the naive H chain isotype, *IgD*, and B cells that expressed isotype-switched *IgG1* (19%) were also present (Figure 1C). Additional evidence for an active intratumoral B cell response was indicated by expression of B cell activation markers *CD40* (47%), *CD86* (12%), *HLA-DRA* (99%), and *HLA-DQA1* (93%) (Figure 1C and Supplemental Figure 2E). Germinal center B cells were also represented, as assessed by their expression of *BCL6* (4%) and *AICDA* (1%) (Figure 1C and Supplemental Figure 2E). In contrast, B cells expressing the reported inhibitory markers *HAVCR1*, *TIGIT*, and *PD1* (9), or immunosuppressive cytokines *IL10* (0.8%) and *IL35* (*IL12A* 2% and *EBI3* 5%) (10, 11), were rarely detected (Supplemental Figure 2E). PCs (*n* = 1,984) were identified by the markers *J chain*, *PRDM1*, and *CD138* and high expression of Ig H and L chains (Figure 1C). Examination of cell trafficking receptors’ expression revealed that both B cells and PCs expressed the mucosal homing receptor integrin α_4_β_7_ (*ITGA4*, *ITGB7*) (12), and PCs expressed the additional mucosal homing receptor *CCR10* (13), but not the intestinal homing receptor *CCR9* (Supplemental Figure 2E), verifying their pancreatic mucosal origin. Thus, PDAC tumors contained activated B cells and terminally differentiated PCs.

We further obtained histopathology images from the 4 resected PDAC samples on which we have performed scRNA-Seq. We observed the presence of multiple TLS-like structures in each tumor sample, with TLSs typically located in the tumor border (Supplemental Figure 3). This observation supports that the B and T cell response revealed by scRNA-Seq may take place inside TLSs.

### Igs from PCs in PDAC featuring isotype switching, somatic hypermutation, and clonal expansion.

**Figure 2:**
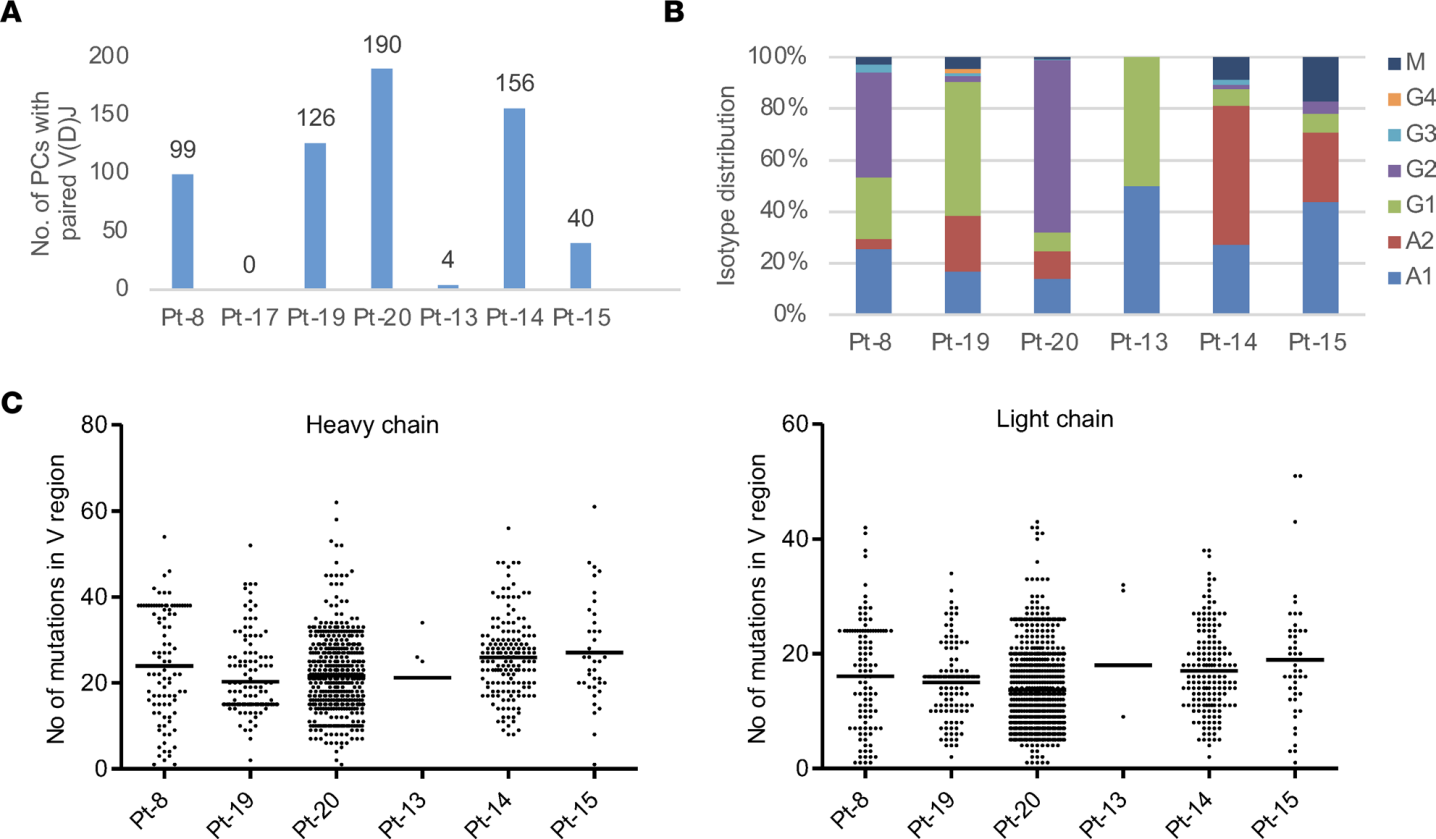
Identification of isotype-switched and somatically hypermutated Igs by sequencing of PCs from PDAC. (**A**) Numbers are shown of PCs in which paired H and L chains were sequenced in each patient. The distribution of Ig isotypes (**B**) and the number of somatic mutations in V regions (**C**) in PCs from individual patients are determined. Individual data point and mean are shown in **C**.

**Figure 3:**
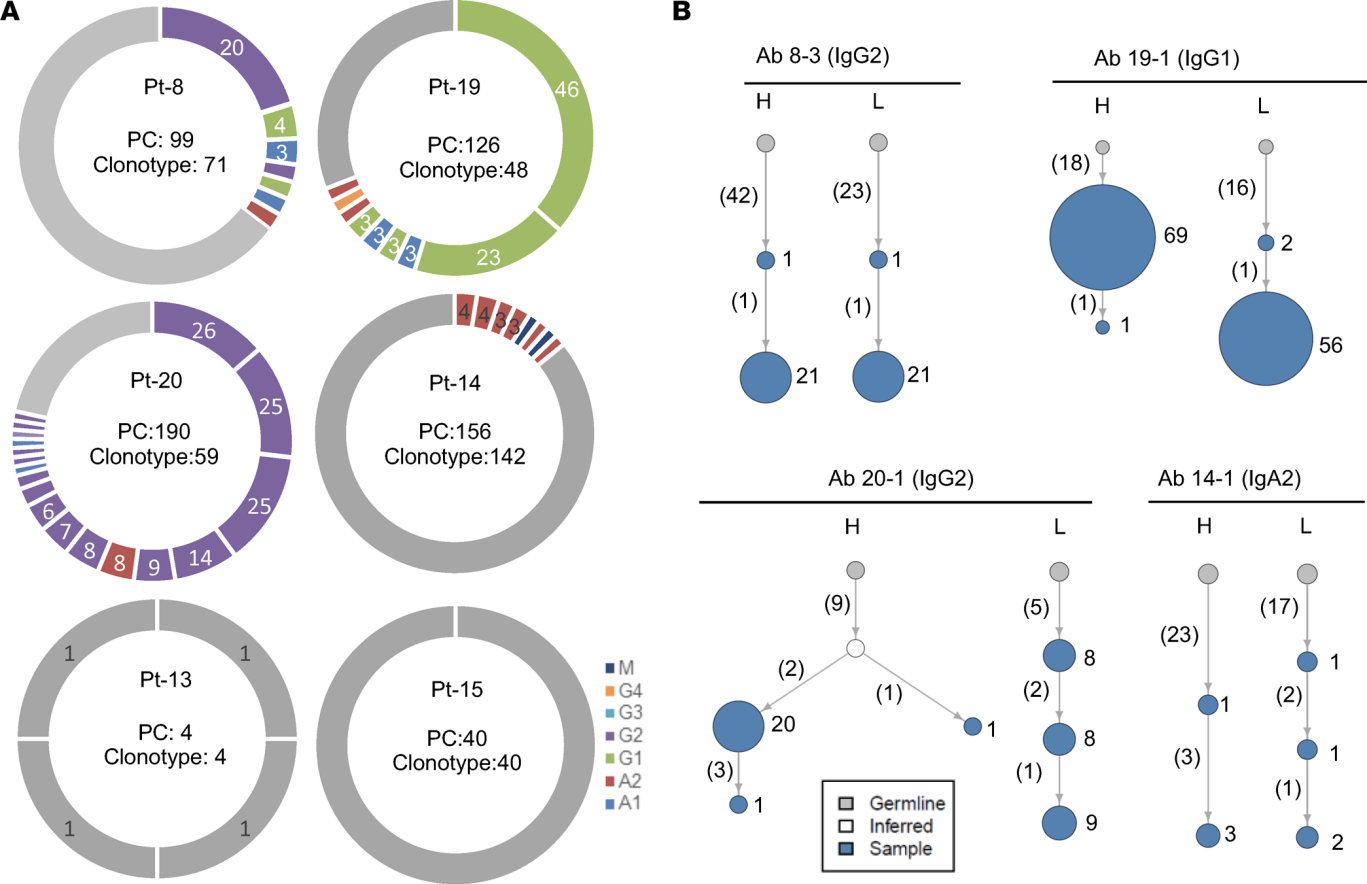
Identification of clonal expanded PCs from PDAC. (**A**) The PCs with paired H and L chains and their clonotype distribution are shown for each patient. PCs within expanded clones are labeled with colored doughnuts, with colors indicating isotypes. Each slice represents 1 clone, with clone size proportional to slice size. Single-clone PCs are pooled and labeled with gray color. (**B**) Examples are presented of antibody lineage evolution within expanded PC clones for individual patients. Clone size is indicated by the size of the node (not scaled) and labeled by numbers on the right. The number of somatic mutations in the V region is shown in parentheses.

A total of 615 PCs with paired H and L chains were detected in 6 PDAC samples by Ig sequencing (Figure 2A). The sample from Pt-17 had no detectable PCs or B cells and was excluded from the analysis. There were additional PCs with only H or L chains being sequenced (Supplemental Figure 4A), likely caused by insufficient sequencing depth. A minority of PCs (0%–17%) expressed the IgM isotype, while the majority had undergone class switching to the IgG1, IgG2, IgA2, or IgA1 isotypes (Figure 2B). For light chains, the frequently used constant regions were *IGKC*, *IGLC2*, *IGLC1*, and *IGLC3* (Supplemental Figure 4B). Analyzing SHM in the V regions revealed an average of 23 and 15 mutations in the H and L chains, respectively (Figure 2C). These rates of SHM are comparable to those in antibodies induced by viral infections (14). Expanded PC clones were identified in 4 of the 6 PDAC samples, with clone sizes ranging from 2 to 46 (Figure 3A). Strikingly, in patients 19 and 20, more than half of the Ig-paired PCs were products of clonal expansion. Analysis of the evolution of V region SHM sequences revealed that most PCs originated from single expanded nodules, although additional lineage evolutions were present (Figure 3B and Supplemental Figure 4C), consistent with typical germinal center reactions. Fewer B cells with paired Igs were sequenced (*n* = 473), likely due to lower Ig expression, which yielded only 4 small, expanded clones (clone size 2-3). Therefore, PCs in PDAC likely arose from germinal center reactions in the PDAC stroma.

### Antibodies from PCs binding to intracellular self-antigens.

**Figure 4:**
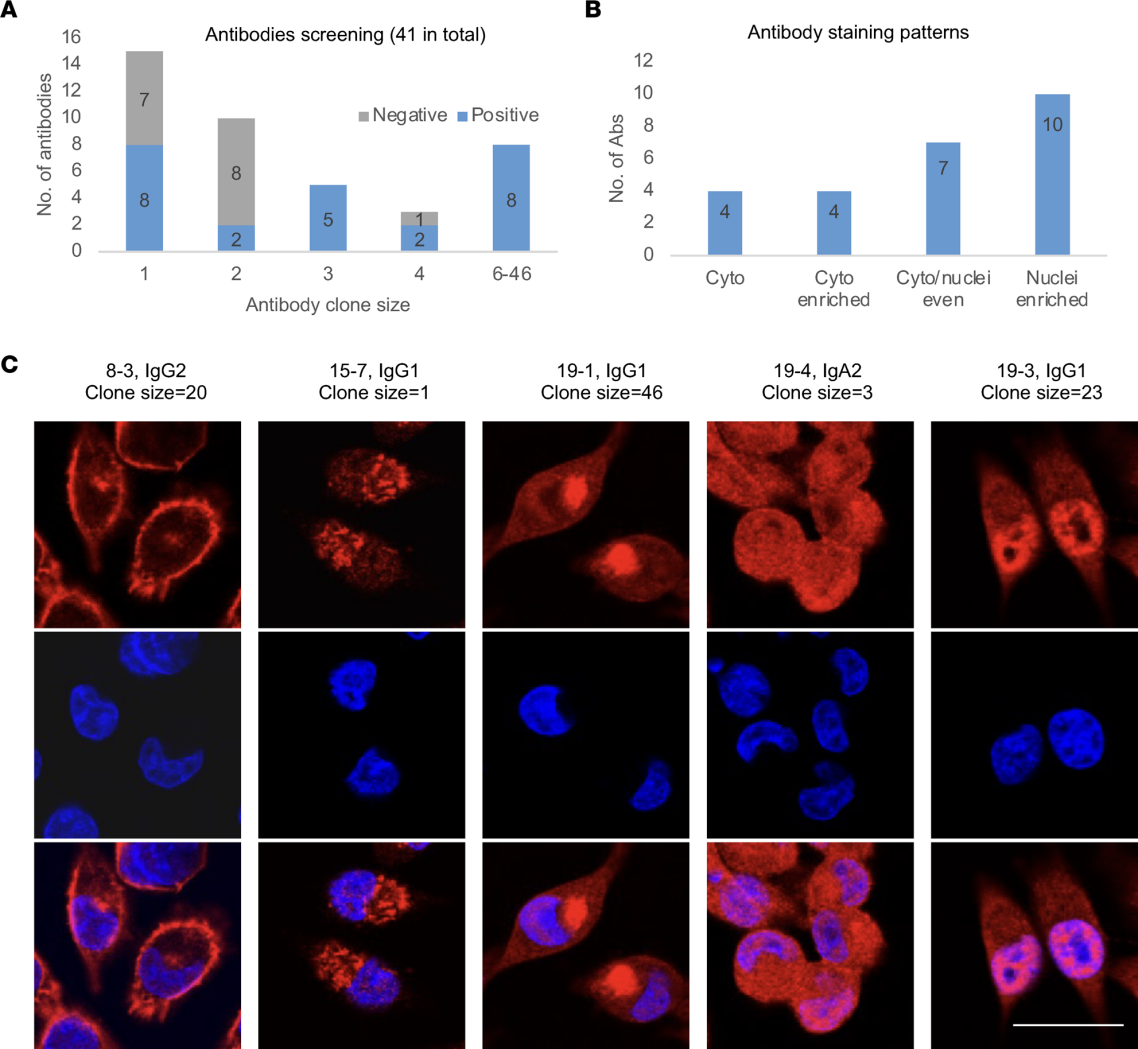
Recognition of intracellular antigens by recombinant antibodies from expanded PCs of PDAC tumors. (**A**) Summary of the results of staining various cell lines with 41 recombinant antibodies based on scRNA-Seq of PCs and B cells from 6 PDAC tumors, distributed according to clone sizes. (**B** and **C**) The distribution and examples are shown of 4 distinct antibody staining patterns: cytoplasmic (Cyto, e.g., 8-3 and 15-7), cytoplasmic enriched (Cyto enriched, e.g., 19-1), evenly distributed in cytoplasmic and nuclei (Cyto/nuclei even, e.g., 19-1), and nuclei enriched (e.g., 19-3). Examples of positive antibody staining (red) in MiaPaca2 cell line, costained with DAPI (blue). The experiments were repeated 3 times. Original antibody isotype and clone size are indicated. Scale bar is 25 μm.

**Figure 5:**
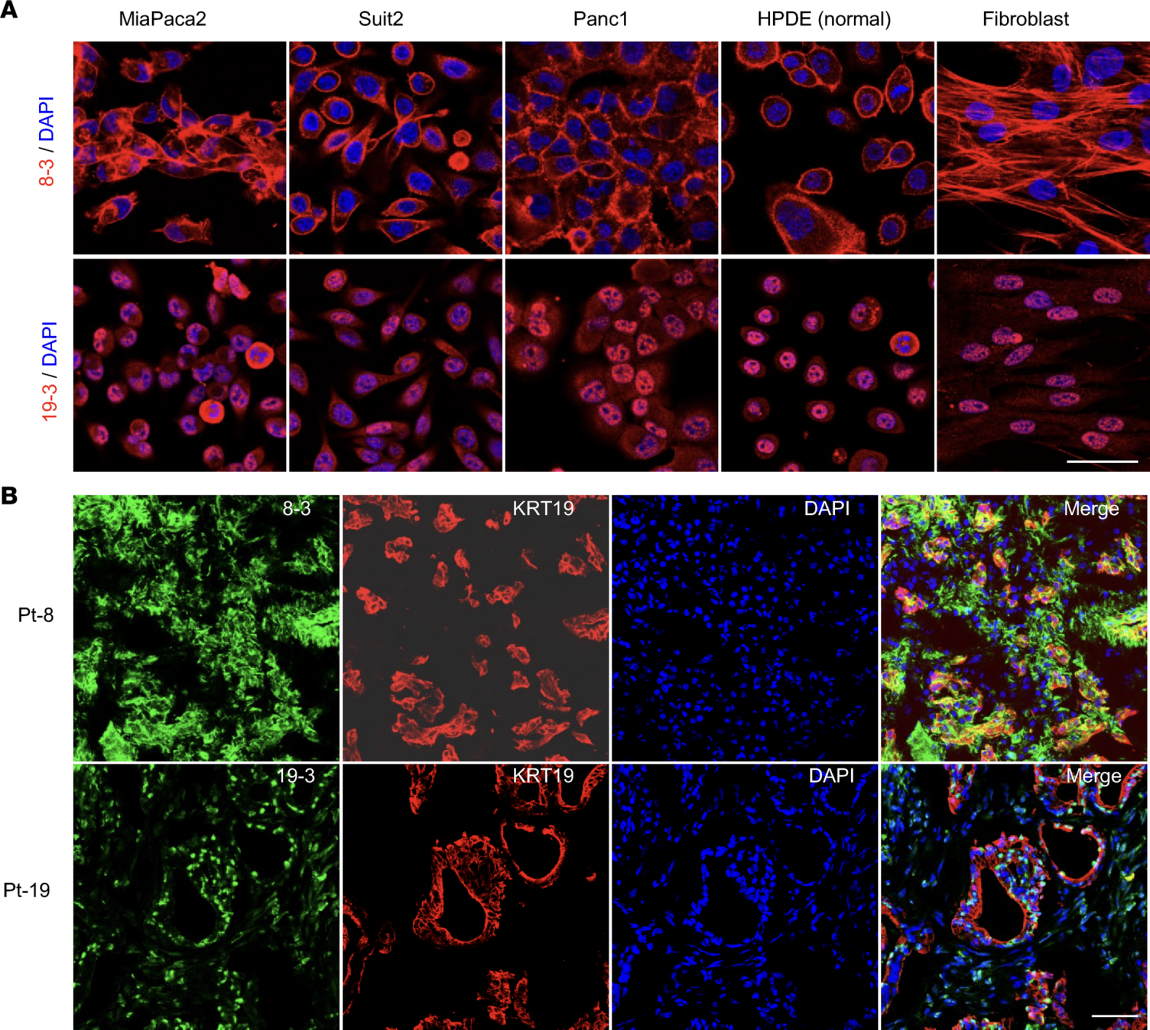
Recognition of intracellular antigens shared by normal and cancer cell lines and tissue by recombinant antibodies 8-3 and 19-3. (**A**) The recombinant antibodies, 8-3 and 19-3 (red), respectively, were used for staining different PDAC and noncancer cell lines, along with nuclear staining with DAPI (blue). (**B**) Antibodies 8-3 and 19-3, respectively, were used to stain the PDAC tumors from which their PCs were derived. Staining was also done with anti-KRT19 antibody to demonstrate the cancer cells. The experiments were repeated 3 times. Scale bars in **A** and **B** are 50 μm.

Forty-one antibodies with paired H and L chains from the 6 patients with PDAC were selected for recombinant antibody synthesis. These antibodies included the most expanded PC cell clones, as well as some single-clone PC and B cells, and represented the products of 235 PCs and 12 B cells (Figure 4A and Supplemental Table 2). Immunofluorescence staining of human PDAC cell lines and tumors was used to screen the reactivity of the recombinant antibodies. Twenty-five of the 41 antibodies showed positive binding to PDAC cell lines, with the expanded clones showing more frequent binding (Figure 4A). Reactive antibodies were identified in each of the 6 patients with PDAC (Supplemental Table 2 and Supplemental Figure 5). Recombinant antibodies reacted with antigens in all subcellular locations, including cytoplasmic (e.g., 8-3 and 15-7), cytoplasmic enriched (e.g., 19-1), both cytoplasmic and nuclear (e.g., 19-4), and nuclear enriched reactivities (e.g., 19-3 and 20-1) (Figure 4, B and C, and Supplemental Figure 5). We did not identify any antibodies staining cell surface antigens. Antibodies derived from a single patient’s PDAC tumor could exhibit diverse staining patterns (for example, 19-1 and 19-3), indicating the occurrence of an adaptive immune response to multiple intracellular antigens in the same patient. All the reactive antibodies could stain multiple PDAC cell lines with a similar staining pattern, as well as the nontumor human pancreatic ductal cell line, HPDE, and human fibroblasts (Figure 5A and Supplemental Figure 6). When staining PDAC tumors from which antibody sequences were derived, the antibodies bound both to cancer cells and to stromal cells (Figure 5B). Thus, nonmutated, non–cancer cell–specific intracellular antigens drive common humoral immune responses in human PDAC.

### Identification of antigens driving PC cell response in PDAC.

**Figure 6:**
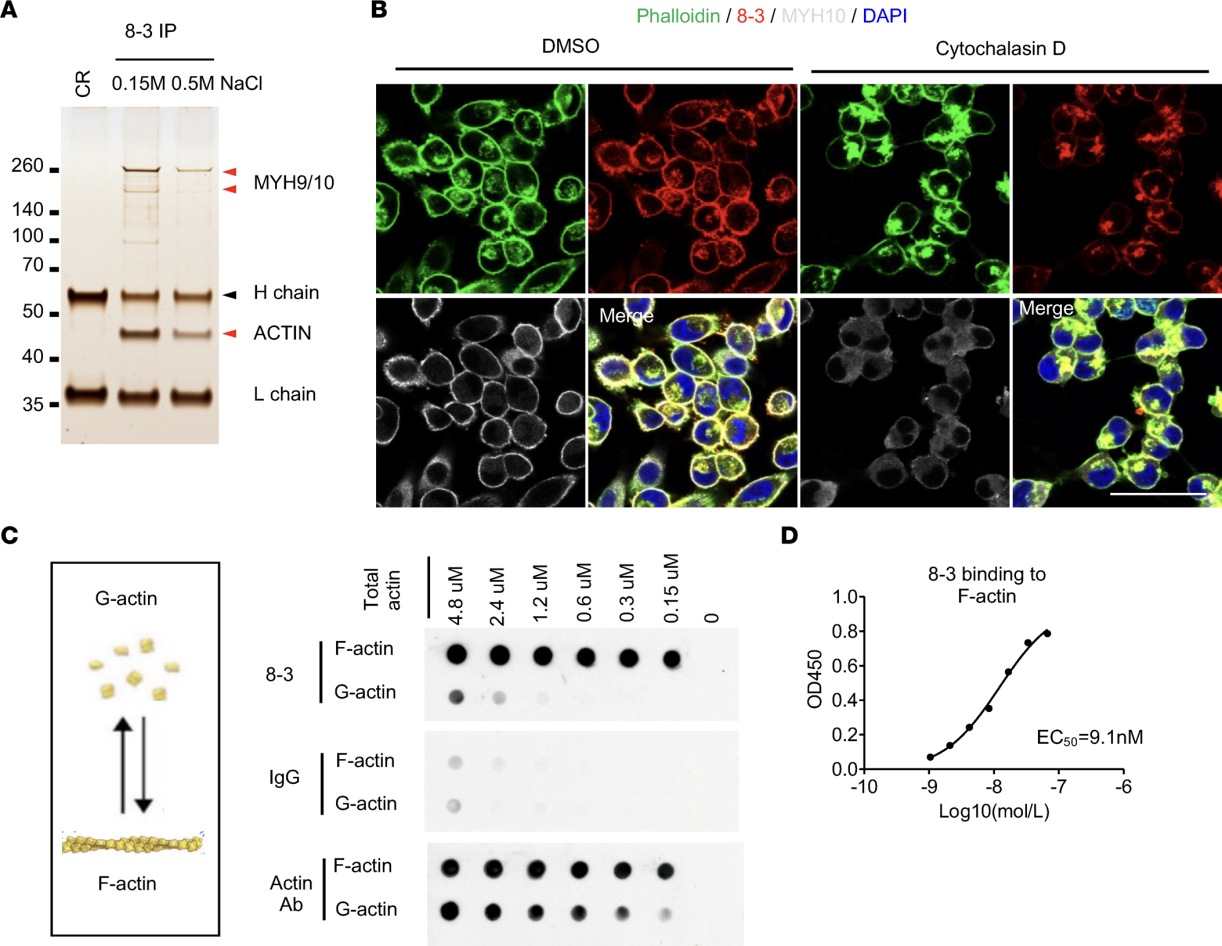
Identification of F-actin as antigen in PDAC. (**A**) Proteins that were immunoprecipitated by antibody 8-3 from lysates of MiaPaca2 cells were separated by SDS-PAGE and visualized by silver staining. Two bands were identified by mass spectrometry (red arrowheads). (**B**) MiaPaca2 cells were costained with 8-3, phalloidin, and an antibody specific for MYH10 after 30-minute treatment of the cells with DMSO or cytochalasin D. (**C**) Dot blot assay is shown of the reaction with F-actin or G-actin of 8-3, isotype control IgG, and a commercial Actin-specific antibody (Actin Ab). (**D**) The binding of incremental concentrations of 8-3 to F-actin was measured by ELISA. Each experiment was repeated at least twice. Scale bars in **B** are 50 μm.

Three PDAC antigens were identified by using antibody-mediated immunoprecipitation from MiaPaca2 cell lysates, followed by mass spectrometry analysis. Antibody 8-3, which was derived from the most expanded PC clone (clone size 20) from patient 8, recognized a cortical structure in MiaPaca2 cells (Figure 4C). This antibody immunoprecipitated a major protein band of approximately 45 kDa, which was identified as Actin by mass spectrometry, as well as the Actin-associated proteins, MYH10 and MYH9 (Figure 6A). Antibody 8-3 colocalized with filamentous actin (F-actin) in the cell cortex, and its antigen relocalized to perinuclei foci after treatment with the Actin-destabilizing drug, cytochalasin D (Figure 6B). Knockdown of Actin with siRNA reduced staining by antibody 8-3, but not staining by an MYH10-specific antibody (Supplemental Figure 7A), indicating that Actin was the target of antibody 8-3. This antibody was verified to bind to polymerized F-actin, but not monomeric globular actin (G-actin), using in vitro actin polymerization and depolymerization assays (Figure 6C). We developed an F-actin–specific ELISA and measured the EC_50_ of antibody 8-3 for F-actin to be 9.1 nM, a relatively high affinity/avidity that was probably a result of SHM (Figure 3B and Figure 6D). Sequencing the Actin genes (*ACTB* and *ACTG1*) from the tumor organoid derived from patient 8, the source of the PCs encoding antibody 8-3, showed no mutations in the protein-coding regions, verifying the self-antigen nature.

**Figure 7:**
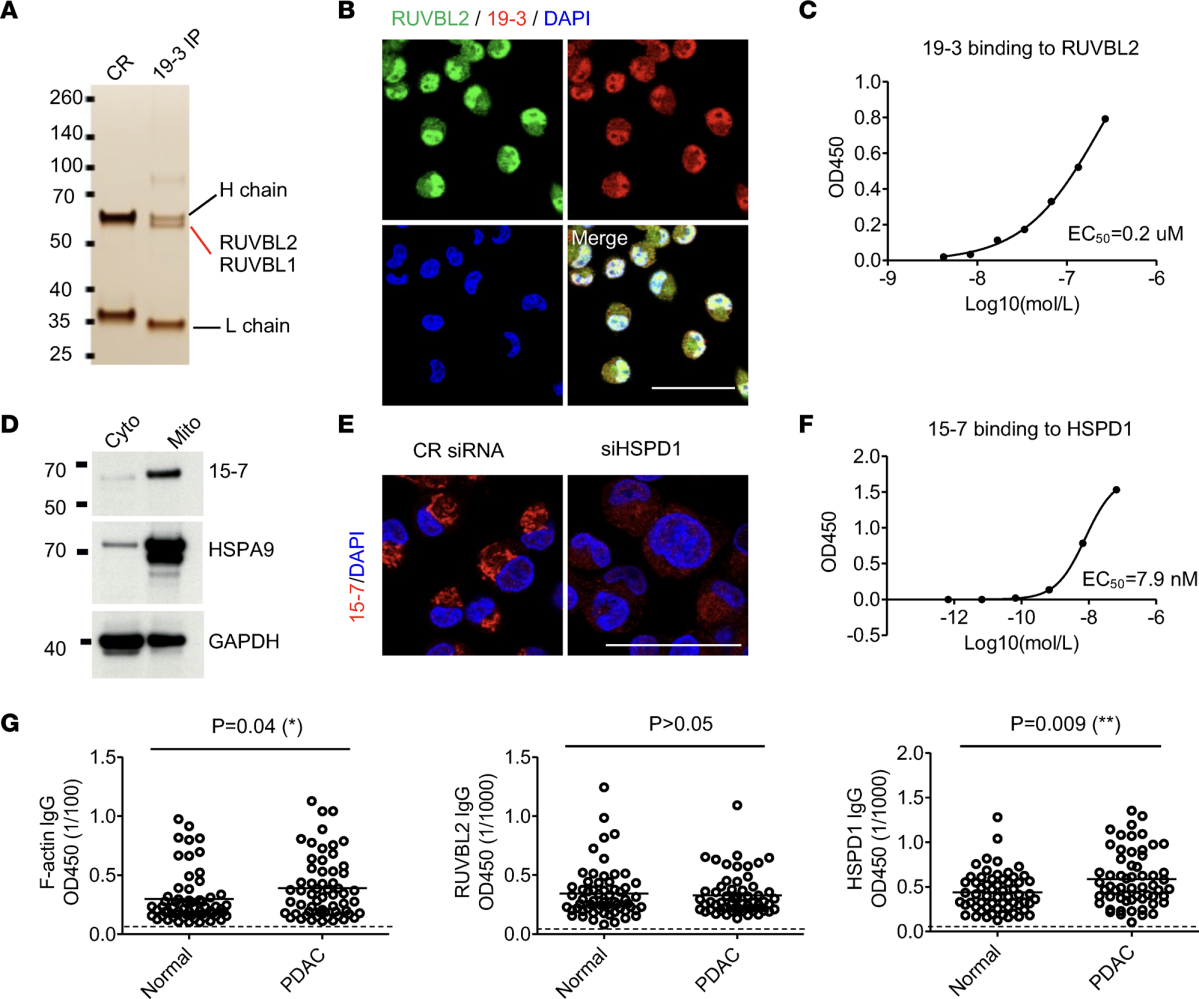
Identification of RUVBL2 and HSPD1 as antigens in PDAC. (**A**) Proteins that were immunoprecipitated by recombinant antibody 19-3 from lysates of MiaPaca2 cells were separated by SDS-PAGE and visualized by silver staining. The proteins, RUVBL1 and RUVBL2, were identified by mass spectrometry. (**B**) MiaPaca2 cells were costained with 19-3 and an antibody specific for RUVBL2. (**C**) The binding of incremental concentrations of 19-3 to RUVBL2 was measured by ELISA. (**D**) Western blot analysis of recombinant antibody 15-7 detection of cytoplasmic proteins (Cyto) and mitochondria enriched proteins (Mito) is shown. (**E**) Immunofluorescence staining of MiaPaca2 cells by 15-7 and an antibody specific for HSPD1 is shown. (**F**) The binding of incremental concentrations of 15-7 to HSPD1 was measured by ELISA. (**G**) The serum IgG titers of normal individuals (*n* = 61) and PDAC patients (*n* = 59) to F-actin (sera diluted 1:100), to RUVBL2 (sera diluted 1:1,000), and to HSPD1 (sera diluted 1:1,000), respectively, were measured by ELISA. Background ELISA signal is indicated by dashed line. Individual data and mean are shown. The nonparametric *t* test was used for comparison. Each experiment was repeated at least twice. Scale bars in **B** and **E** are 50 μm.

Similarly, antibody 19-3, the second most expanded PC clone in patient 19 (clone size 23), immunoprecipitated a protein complex of RuvB like AAA ATPases 1 and 2 (RUVBL1 and RUVBL2), as determined by mass spectrometry (Figure 7A). Knockdown of RUVBL2 reduced antibody 19-3 binding in the MiaPaca2 cell line (Figure 7B and Supplemental Figure 7B). This antibody recognized recombinant RUVBL2 but not RUVBL1 protein, with an EC_50_ of 0.2 μM (Figure 7C and Supplemental Figure 7C), confirming that antibody 19-3 recognizes RUVBL2.

Antibody 15-7, which was derived from a single PC clone in the tumor from patient 15, costained with antibodies specific for the mitochondrial markers, COX4I1 and HSPA9 (Supplemental Figure 7D). This antibody recognized a mitochondrially enriched protein band with an apparent molecular weight of 60 kDa (Figure 7D), which was identified as heat-shock protein D1 (HSPD1) by mass spectrometry. HSPD1 was verified to be the antigen of antibody 15-7 by immunofluorescence colocalization in MiaPaca2 cells, siRNA knockdown, and recombinant protein binding, with an EC_50_ of 7.9 nM (Figure 7, E and F, and Supplemental Figure 7D).

In summary, our identification of 3 self-antigens, including antigens for 2 antibodies from highly expanded PC clones, indicates that widely expressed, intracellular self-antigens may be targets of humoral immune responses in human PDAC.

### The frequent occurrence of antibody responses to F-actin and HSPD1 in patients with PDAC.

We collected plasma samples from 59 patients with PDAC and 61 healthy donors and measured IgG titers to F-actin, RUVBL2, and HSPD1, respectively (Supplemental Table 3). Demographics, such as sex, age, and race, did not significantly affect antibody concentration. Interestingly, plasma from healthy donors contained detectable levels of IgG responses to these 3 antigens. In PDAC, the IgG F-actin and HSPD1 antibody titers were significantly higher than those in normal individuals (Figure 7G and Supplemental Figure 8A). IgG F-actin antibody titers were significantly reduced in patients who had received neoadjuvant therapy, but this was not observed for either HSPD1 or RUVBL2 antibody titers (Supplemental Figure 8B), suggesting that B cell responses for different antigens may have different sensitivities to cytotoxic chemotherapy. These antibody responses did not correlate with other pathological features such as tumor stage, size, or grade. Thus, patients with PDAC likely promote autoantibody responses that preexist in healthy donors.

## Discussion

PDAC is generally considered to be one of the most immunologically “silent” carcinomas and to be resistant to current immunotherapies for this reason. Reports, however, of TLSs in PDAC (2–4), of neoantigens with homology to infectious disease-derived peptides in long-term survivors of PDAC (6), and signs of improved anti-PDAC immunity in metastatic lesions of patients with PDAC after inhibition of CXCR4 (5) all point to a need for a deeper understanding of the relationship between this cancer and the immune system. The antigens driving the B cell response are more readily identified by the Ig product of the B cells and PCs. Thus, we chose to interrogate the Ig products of intratumoral B/PCs in PDAC.

Our scRNA-Seq analysis of CD45^+^ immune cells in primary PDAC samples indicated active intratumoral immune reactions, such as the presence of effector CD8^+^ T cells expressing *PRF1*, *GZMB*, and *IFNG* and effector CD4^+^ T cells expressing *TNF* and *IFNG*. We were able to verify the 4 resected PDAC tumor samples as being microsatellite stable, representing the majority of PDAC (15). The presence of CD8^+^ T cells expressing inhibitory markers such as *LAG3*, *TIGIT*, *KLRC1*, and *PD-1*, and *FOXP3*^+^ regulatory CD4^+^ T cells may prevent effective T cell–mediated tumor control in PDAC. The presence of effector T cells in our cohort is consistent with previous studies of human and mouse autochthonous PDAC immune microenvironments in which activated, effector T cells were found (16). A previous preprint study with single-cell sequencing and meta-analysis in PDAC also identified cytolytic CD8^+^ T cells and their association with improved patient outcomes (17). Current T cell checkpoint therapy (such as anti–PD-1 antibody) has not been effective in PDAC (1); thus, additional immunotherapy will be needed to enhance the preexisting T cell activity (5).

In addition, scRNA-Seq identified CD4^+^ T cells with the Tfh phenotype by their expression of *BCL6* and *CXCR5* and germinal center B cells by their expression of *BCL6* and *AICDA*. B cells transcribed activation markers such as *CD40*, *CD86*, and *MHC-II* but rarely expressed the reported inhibitory markers, such as *HAVCR1* (9), *IL10* (11), or *IL35* (10). The presence of a B cell response is further supported by Ig sequencing that identified isotypically switched, somatically hypermutated, and expanded PC clones. The high frequency of PCs (7.4%) and presence of multiple expanded PC Ig clones indicate that the identified PCs are unlikely to be coming from bloodborne circulating PCs, which typically are less than 0.2% (18). This frequency of PCs and B cells was also observed in flow cytometry analysis. Both B cells and PCs expressed the mucosal homing receptor integrin α_4_β_7_ (12), and PCs expressed the additional mucosal homing chemokine receptor *CCR10* (13), further supporting the pancreatic tissue origin of cells. The presence of TLS in PDAC stroma from the paired histology images further support the likelihood of integrated B and T cell responses, presumably occurring in germinal centers.

There are a few reports on identifying antigens inducing humoral responses in PDAC. Most of those studies are done by using serologic profiling of PDAC patient serum binding to candidate antigens, PDAC cell lysates, or a peptide/protein array. For example, some patients with PDAC were reported to have a plasma antibody binding to MUC-1 (19), p53 (20), or KRAS (21) using candidate antigen screening. Three studies used serum from patients with PDAC probing cell lysates separated by 2-dimensional Western blot electrophoresis and detected binding to intracellular antigens such as vimentin (22), PGK1 (23), and laminin (24). A peptide library screening with serum from patients with PDAC identified a low-frequency binding to UBR2 peptide in 2 out of 40 PDAC samples (25). A comprehensive seromic profiling of patients with PDAC (*n* = 60) with a human whole-genome recombinant protein array has identified 28 antigens with increased immunogenicity compared with healthy controls (*n* = 53) (26). Most of the identified antigens are intracellular proteins, including the top-ranked candidates, such as MAPK9, NR2E3, C6orf141, and ROR2 (26). The frequency of patients with PDAC with serum antibodies against those identified antigens is generally low (less than 20%). The drawback of those serologic approaches is that it is uncertain whether the B cell responses occurred inside the tumor, or whether the antibodies displayed somatic hypermutation, which depends on help from CD4^+^ T cells. In addition, those assays did not address the antigen hierarchy of B cell responses in PDAC.

Our study is the first to our knowledge using Ig single-cell sequencing and recombinant antibody screening to study intratumor B cell and PC antigen reactivity in PDAC. This provides valuable insights not only into intratumor B cell reactions but also into antigen dominance hierarchies, as revealed by PC clonal size. While we were unsuccessful in our initial intent to discover tumor-specific B cell antigens, the finding of Igs from PCs frequently binding to widely expressed, nonmutated intracellular self-antigens in human PDAC is intriguing. Our screening in PDAC identified most antibodies as recognizing intracellular antigens that were shared by multiple PDAC cell lines, normal epithelial cells, and fibroblasts. This conclusion was validated by the identification of 3 self-antigens, F-actin, RUVBL2, and HSPD1, as targets of these humoral immune responses. Sequencing of *Actin* genes from organoids derived from the same patient did not reveal mutations, ruling out a neoantigen origin. Thus, the B cell response to F-actin is driven by self-antigen. Though antibody responses to F-actin, RUVBL2, and HSPD1 have been reported in other cancers (27, 28), they are here identified to induce B cell response in PDAC. It is worth noting that our identified antibody (Ab 8-3) reactive to F-actin is conformationally specific, as the antibody does not recognize monomer G-actin or denatured Actin in Western blot. Therefore, this antigen will not be identified using typical protein array approaches. We did not exclude that additional unscreened small or single PC clones may recognize tumor-specific antigens or surface antigens. However, the most expanded PC clones screened in this study all recognized intracellular antigens expressed by both tumor and nontumor cell lines. The majority of the previous antigens identified by PDAC serologic profiling studies also are intracellular. Taken together, intracellular self-antigens are likely driving the dominant B cell response in PDAC.

It is intriguing how B cell response to such intracellular antigens is generated in PDAC. The 7 patients with PDAC enrolled in this study have no records of autoimmune disease or pancreatitis. In this context, it is worth noting that 20% of naive B cells in human peripheral blood are reported to be autoreactive (29), which is consistent with our detection of autoantibody responses to those antigens in healthy donors. Self-reactive B cell responses are likely promoted in PDAC because of continuous exposure to intracellular self-antigens through cell death and chronic inflammation, reminiscent of the antibody responses in autoimmune diseases. Antibody responses to F-actin, RUVBL2, and HSPD1 have been also reported in various autoimmune conditions (30–32). Previously, immunization with mouse syngeneic embryonic stem cells reduced mouse pancreatic tumor progression, indicating self-antigens likely drive such immune response (7, 8). Additionally, a recent study reported that the self-protein matrix metallopeptidase 14 was a major autoantigen in human ovarian cancer, and some Igs likely originated from germline-encoded autoreactive B cells (33). Thus, the B cell response to self-antigens may be a common feature of cancer.

Our analysis of plasma titer to F-actin, RUVBL2, and HSPD1 did reveal that patients with PDAC have significantly higher titers to F-actin and HSPD1 as compared with a group of healthy donors (Figure 7G). Our analysis did not reveal a significant correlation with tumor-associated pathological factors, although this may be partially due to our relatively small cohort of patients. Our studies can be potentially strengthened by analyzing serial tumor biopsies and plasma obtained during tumor progression or response to therapy, such as CXCR4 inhibition, thus providing more dynamic pictures of B cell response and antibody titer changes.

Our study was limited by its small sample size and variable composition of immune cells in each sample. This variation is likely due to sampling, as we have noted that TLSs, which will contain the germinal centers, are typically found in the border of the tumor and stroma and therefore subject to sampling biases. Whole-tumor imaging and imaging-guided laser dissection will provide a more consistent sampling of TLSs. Also, our Ig sequencing revealed that for PC-rich samples, such as Pt-20, the ratio of PCs with paired H and L chains sequenced was low. This may be a result of the elevated representation of the most UMI-rich cells in the library, which outcompete lower expressing clones during sequencing. It is possible that additional sequencing depth could yield better recovery of both H and L chains.

One open question from this study is whether the B cell response to intracellular self-antigens affects PDAC progression. Given the generally positive correlation of a B cell response with a favorable outcome in PDAC (2–4), we speculate that those autoreactive B cell responses may indirectly have an antitumor role. While antibodies binding intracellular antigens cannot directly target live tumor cells, these antigens can be exposed to antibody binding during cancer cell death, such as necrosis, and potentially induce inflammation through complement activation and Fc receptor binding. Moreover, we expect that B cells binding such self-antigens will internalize and engage in antigen presentation to CD4^+^ T cells. F-actin has been reported to promote antigen presentation through the F-actin receptor Clec9A on dendritic cells in a breast cancer model (34). Further understanding of the B cell response in PDAC may provide opportunities for the modulation of cancer immunotherapy. In any event, the finding of T cell and B cell responses in PDAC tumors indicates that an adaptive immune response is occurring in the tumor microenvironment, which may be exploited for tumor control.

## Methods

### Study design.

This study is designed to study B cell response and identify B cell–targeted antigens in human PDACs. This study consists of 1) scRNA-Seq of immune cells infiltrating human PDAC and Igs of cells and PCs, 2) synthesizing of recombinant antibodies from Ig sequencing, 3) screening of recombinant antibodies reactivities toward PDAC cell lines and tumor tissues, and 4) identification of antigens. This pipeline was used to study 4 resected and 3 FNA naive-treated PDAC samples. The antibody synthesizing and screening aim to profile the most expanded PC clones in PDAC to get a better picture of B cell reactivity in human PDAC.

### Human samples.

All human samples were obtained with written consent and IRB approval. Four resected PDAC specimens and 3 FNA (2 passes each) specimens were obtained from Northwell Health. Archived plasma samples from patients with PDAC (*n* = 59) were received from Northwell Health. Plasma samples from healthy donors (*n* = 61) were obtained from volunteer donors or purchased from commercial resources (Innovative Research and BioChemed Services). Microsatellite instability status of resected PDAC samples was determined by immunohistochemistry staining of MLH1, MSH2, MSH6, and PMS2, performed and interpreted by pathologists from Northwell Health. Histopathologic hematoxylin and eosin images were obtained from the Northwell pathology department and acquired by Leica Aperio slide scanner. All patient samples were deidentified.

### Cell lines and culture.

Human PDAC cell lines MiaPaca2, Suit2, Panc-1, and foreskin fibroblast (BJ-hTert) were obtained from Cold Spring Harbor Laboratory cell validation center and had been validated by short tandem repeat profiling. The normal human pancreatic ductal epithelial cell line (HPDE) was obtained from Adrian Krainer’s laboratory (Cold Spring Harbor Laboratory), originally purchased from Kerafast. All cell lines had been tested mycoplasma free and cultured in the conditions recommended by ATCC or the manufacturer.

### Tumor sample preparation and single-cell sequencing.

Fresh tumor samples were prepared and used on the same day. A small portion of the resection tumor was snap-frozen for histology. The remaining human PDAC tumor samples were digested into single cells using collagenases, and about 10% of single-cell digest was used for tumor organoid culture as described (35). Briefly, the PDAC tissue was minced into small pieces using sterile scalpels, then digested with 5 mg/mL collagenase XI, 10 μg/mL DNase I, and 10.5 μM ROCK inhibitor Y-27632 (all from MilliporeSigma), in 37°C water bath for 15 minutes under shaking (35 rpm). The cell suspensions were filtered through a 100 μm cell strainer (Corning), then lysed with red blood cell lysis buffer. About 10% of single cells were used for organoid culture according to the established protocol (35). For flow cytometry analysis and sorting, the cell suspension was blocked with human Fc blocker (BioLegend, 422302), then stained with antibody cocktails containing CD45-APC-Cy7 (BioLegend, 368516), EpCAM-Percp-Cy5.5 (BioLegend, 324214), CD19-PE (BioLegend, 302254), CD38-APC (BioLegend, 303510), and DAPI. Live immune cells were sorted by EpCAM^–^CD45^+^ on a Sony SH800 cell sorter. About 10,000 immune cells from each sample were barcoded with 10x Genomics Chromium Single Cell 5′ Gene Expression kit (10x Genomics 1000020), and both whole-transcriptome and Ig (10x Genomics 1000016) sequencing libraries were prepared according to the 10x Genomics manual. Each sample was processed into indexed libraries immediately upon receipt and stored frozen until 2–3 samples could be sequenced together. Sequencing was carried out on a combination of Illumina NextSeq500 (for gene expression Ig) and MiSeq (for Ig) instruments.

### Sequencing data analysis.

The scRNA-Seq and Ig reads were aligned and quantified using the Cell Ranger pipeline (10x Genomics, version 6.0.0). Dead cells and cells with the number of genes expressed less than 200 were also filtered out. ScRNA-Seq data were normalized, logarithm-transformed, and scaled. Doublets were removed using R package Scrublet. Principal component analysis was run with 50 components using the top 4,000 genes of each sample. The nearest neighbor algorithm was run with 50 principal component analyses and clustered in UMAPs using Leiden clustering. All additional analyses were performed using Loupe Browser (10x Genomics, version 6.1.0) and the Python toolkit Scanpy 1.6.0. Immune cell clusters were defined using the set of markers and UMAP clusters: T cells (CD3^+^, TCR^+^, NCAM^–^), NK cells (CD3^–^, NCAM^+^), B cells (CD20^+^, CD79A^+^, IGKC^lo^), PCs (JChain^hi^, IGKC^hi^), myeloid cells (CD14^+^, CSF1R^+^), mast cells (CPA3^+^, GATA2^+^), fibroblasts (FAP^+^, ACTA2^+^, PDGFRA1^+^), epithelial cells (CK19^+^, CK18^+^, CK5^+^, CK14^+^).

### Ig data analysis.

PC and B cell clusters defined by scRNA-Seq were used to select cell type–specific Ig analysis. In 1 patient (Pt-15), gene expression sequencing data were not available; the PCs were defined by high expression of H chain (UMI ≥ 100). B cell– or PC-specific Igs were exported from the Loupe V(D)J Browser (10x Genomics, version 4.0.0) and analyzed for isotypes and clonotypes. For somatic hypermutation analysis, V region sequences were analyzed by Igblast (NCBI) and visualized by GraphPad Prism 5. For lineage tree analysis, Ig sequences were first analyzed using R packages Change-O and alakazam (36), and the Ig lineages were built using an R package PHYLIP. Finally, lineage trees were visualized using an R package igraph.

### Recombinant antibody production.

The selected antibody V(D)J sequences were synthesized from IDT, then cloned into pFUSEss H and L vectors (InvivoGen) using NEB HIFI assembly kit (5520s). The heavy chains were cloned into human IgG1 or customer-designed human IgG4-His vector. A 6x His tag with a Gly-Ser-Gly linker was inserted before the stop codon of human IgG4 constant region, was synthesized, and replaced the IgG1 in pFUSE-CHIg-hG1 vector. The light chain was cloned into a vector containing IgKC, IgLC2, or custom-made IgLC1 vector, to match the original light chain. Sequence-confirmed antibody vectors were mixed at H/L ratio 1:1.5, then transiently transfected into HEK293T cells (ATCC), cultured in 10% IgG reduced serum. Culture supernatants were collected 4 days later, and antibodies were purified using the Protein G column (Thermo Fisher Scientific, 89956). The antibodies were further concentrated, and the buffer was exchanged into PBS using Amicon 50K centrifugal filter device (MilliporeSigma, UFC805096). The antibody concentration was quantified by NanoDrop (Thermo Fisher Scientific) and human IgG ELISA kit (Mabtech, 3850-1H-6).

### Antibody screening using immunofluorescence staining.

Cells were seeded in the 24-well, glass-bottom cell culture plate (Chemglass, CLS-1812-024), then used 2–3 days later at 50%–80% confluence. Cells were fixed with 4% paraformaldehyde, permeabilized with 0.1% Triton X-100, blocked with 3% FBS, and stained with 10 μg/mL human query antibodies (generated from this study) diluted in PBS with 0.05% Tween 20 (PBST) containing 1% FBS overnight at 4°C. Cells were then washed 3 times with PBST, then stained with fluorescence-conjugated secondary anti-human IgG antibody (Thermo Fisher Scientific, A21090 and A-11013) and DNA dye DAPI. In human tumor section staining, anti-His secondary antibody (BioLegend, 362607) was used to detect IgG4-His primary antibodies. Other antibodies and dyes used were Phalloidin (Thermo Fisher Scientific, A12379), MYH10 (Atlas Antibodies, HPA047541), cytochalasin D (Thermo Fisher Scientific, PHZ1063), Actin (Cell Signaling Technology, 3700S), RUVBL2 (Atlas Antibodies, HPA067966), RUVBL1 (Atlas Antibodies, HPA019947), HSPD1 (Atlas Antibody, HPA050025), HSPA9 (Atlas Antibody, HPA000898), COX4I1 (Atlas antibody, HPA002485), KRT19 (Abcam, ab203445), and human IgG isotype controls (BioLegend, 403502 and 403702). The samples were imaged in Leica SP8 confocal microscope under 40× original magnification. Antibody screening images were acquired using the same confocal setting for isotype control staining, and the gain of query antibodies was reduced if images were saturated in the setting. Images were analyzed using Leica LAS X or NIH ImageJ software.

### Immunoprecipitation and mass spectrometry.

Antibodies were covalently crosslinked to magnetic beads using the Thermo Fisher Scientific Dynabeads Antibody Coupling Kit. The total cell lysate was prepared from MiaPaca2 cell line using TNET lysis buffer (50 mM Tris-HCl, pH 7.5, 150 mM NaCl, 5 mM EDTA and 1% Triton X-100). Dynabeads containing 2–5 μg crosslinked antibodies or isotype control were first blocked with 1% BSA, then incubated with up to 1 mg cell lysate overnight in 4°C in TNET buffer. The beads were separated using a magnetic rack, washed 3 times with TNET buffer (or with increased NaCl concentration), and eluted with 2× SDS under boiling. The immunoprecipitation elute was run in gradient SDS-PAGE gel and stained with a silver staining kit (Thermo Fisher Scientific, 24600).

Distinct gel bands were excised, de-stained, reduced with 3 mM TCEP and alkylated with 10 mM CEMTS, and then digested with trypsin. Eluting peptides were ionized and transferred into an Exploris Orbitrap mass spectrometer (Thermo Fisher Scientific). Spectral data were searched against the human database and a database of common contaminants. M-oxidation and N/Q-deamidation were set as variable modifications. Peptide-spectral matches were filtered to maintain FDR < 1% using the Percolator.

### siRNA knockdown.

Cells were transfected with control or siRNA at 20–60 μM, cultured for 3 days, and used for immunofluorescence staining. siRNA against human ACTB (SASI_Hs01_00204238, SASI_Hs01_00204239), MYH10 (SASI_Hs01_00072460, SASI_Hs02_00340636), MYH9 (SASI_Hs01_00197338, SASI_Hs01_00197339), RUVBL2 (GATGATTGAGTCCCTGACCAA, GAAGATGTGGAGATGAGTGAG), and control (GGATGTAAGTGGGAAAGTGGA) were purchased from MilliporeSigma. siRNA targeting HSPD1 (L-010600-00-0005) was purchased from Horizon Discovery.

### Actin polymerization or depolymerization and dot blot assay.

Human nonmuscle Actin (Cytoskeleton, APHL99) was polymerized or depolymerized according to the manufacturer’s protocol. Briefly, Actin was first diluted into 0.4 mg/mL in G-buffer (5 mM Tris-HCl, 0.2 mM CaCl_2_, 0.2 mM ATP), and kept on ice for 1 hour. For polymerization, 10% polymerization buffer (500 mM KCl, 20 mM MgCl_2_) with 1 mM final ATP and 5 μM phalloidin (MilliporeSigma, P2141) was added to the actin and incubated at room temperature for 1 hour. For depolymerization, 5 μM latrunculin B (MilliporeSigma, 428020) was added into the Actin with G-buffer and incubated in ice for 1 hour. Actin samples were further diluted into G-buffer or polymerization buffer supplemented with phalloidin or latrunculin B, and serial dilutions of F-actin or G-actin were blotted on nitrocellulose membrane using Bio-Dot microfiltration apparatus (Bio-Rad) according to the manufacturer’s instructions. The membrane was further blocked and incubated with human IgG4 isotype control, Ab 8-3, mouse anti-human Actin antibody (Thermo Fisher Scientific, MA1-140) at 1 μg/mL for 1 hour; washed and detected with HRP conjugated anti-human IgG (Thermo Fisher Scientific, A18811) or anti-mouse IgG (BioLegend, 405306) secondary antibody; and developed by ECL substrate.

### ELISA.

Recombinant proteins RUVBL2 (Creative Biomart, RUVBL2-30950), RUVBL1 (Novus, NBP1-50845), HSPD1 (Origene, TP760396), or BSA control were coated in PBS with 0.25 μg per well in a 96-well ELISA plate (Corning) overnight in 4°C. The plate was blocked with 1% BSA, washed with PBST, incubated with query antibodies or isotype control at 1 μg/mL diluted in 0.1% BSA in PBST for 1 hour, detected with HRP-conjugated secondary antibody (1 μg/mL), and developed by TMB substrate (BioLegend, 421101) for 5–10 minutes. For F-actin ELISA, polymerized F-actin was diluted in polymerization buffer supplemented with 5 μM phalloidin and 1 mM ATP, and coated in the ELISA plate for 1 hour at room temperature, followed by antibody binding and development.

For human plasma studies, the antigens were coated as above, then blocked with 5% milk in PBS. PDAC or healthy donor plasma was diluted 1/100 and 1/1,000 in 0.5% milk in PBST, and each sample was run in duplicates. Positive controls are using antibodies 8-3, 19-3, or 15-7. Background signal was obtained using the higher signal from secondary antibody only and plate coated with BSA. All samples to the same antigen were analyzed at the same time to avoid batch differences. Each ELISA analysis was repeated twice.

### Mitochondria cell fraction and western blot.

For crude mitochondria fraction, the cells were first lysed using low-salt buffer (0.25 M sucrose, 20 mM HEPES-KOH pH 7.5, 10 mM KCl, 1.5 mM MgCl2, 1 mM EDTA, 1 mM EGTA, 1 mM DTT) supplemented with proteinase/phosphatase inhibitors, homologized with a Teflon-glass homogenizer, and then centrifuged at 700*g* for 10 minutes at 4°C to collect the cytosol fraction in the supernatant. The cytosol fraction was further centrifuged at 10,000*g* for 15 minutes at 4°C to collect the mitochondria fraction in the pellet. Same amounts of SDS-reduced cytosol or mitochondria fractions were run in Western blot, then detected with antibodies 15-7, HSPA9, and GAPDH (Cell Signaling Technology, 97166). A separate sample gel was silver stained and cut around 60 kDa for mass spectrometry analysis.

### Histopathology image analysis.

The scanned resected PDAC histopathologic images were opened with ImageScope (Leica), and lymphocyte aggregates bigger than 0.01 mm^2^ size were counted and exported for size measurement using ImageJ (NIH).

### Statistics.

Statistical analysis was performed using GraphPad Prism 5. The plasma IgG data were first tested for normality distribution in GraphPad. Mann-Whitney *t* test was used for a 2-group comparison of non-normal distribution data. Kruskal-Wallis ANOVA test was used for comparisons of 3 or more groups of non-normal distribution data. Fisher’s test was used for demographic factor comparison, and Pearson’s correlation was used for correlation studies. A *P* value less than 0.05 was considered significant.

### Study approval.

All human samples were obtained with written consent at and IRB approval from (TDP-1806, TDP-1905, and TDP-2115) Cold Spring Harbor Laboratory (Cold Spring Harbor, New York, USA) and Northwell Health (Manhasset, New York, USA).

### Data availability.

The sequencing data from this study have been deposited into the NCBI GEO database with accession number GSE238163. Public codes/software was used for the data analysis as described in the paper. The data points in the figures are available in the Supporting Data Values XLS file.

## Author contributions

MY and DTF conceived the study. MY, JP, JTHY, DP, PC, YZ, BH, HP, ANH, DAK, KR, AR, DS, MJW, and DT developed methodology. MY, SS, and PM investigated. MY, JP, YZ, and BH visualized data. DTF acquired funding. MY performed project administration. DTF supervised. MY and DTF wrote the manuscript. YZ, PM, KR, AR, and JP reviewed and edited the manuscript.

## Supplementary Material

Supplemental data

Supplemental table 2

Supporting data values

## Version 1 09/26/2023

In-Press Preview

## Version 2 11/08/2023

Electronic publication
