# Supplementary material for: Plasma cells in human pancreatic ductal adenocarcinoma secrete antibodies against self-antigens
Source: JCI Insight. 2023 Nov 8;8(21):e172449. doi: 10.1172/jci.insight.172449 (PMC10721257; doi:10.1172/jci.insight.172449)
Supplement: Supplemental data [file jciinsight-8-172449-s307.pdf]

Title: Plasma cells in human pancreatic ductal adenocarcinoma secrete antibodies to self-antigens

Authors: Min Yao[1]†, Jonathan Preall[1], Johannes T.-H. Yeh[1], Darryl Pappin[1], Paolo Cifani[1], Yixin Zhao[1], Sophia Shen[2], Philip Moresco[1,3,4], Brian He[1], Hardik Patel[1], Amber N. Habowski [1], Daniel A. King[5], Kara Raphael[5], Arvind Rishi[5], Divyesh Sejpal[5]‡, Matthew J. Weiss[5], David Tuveson[1], Douglas T. Fearon[1,6*]

Supplementary material

Supplementary figures

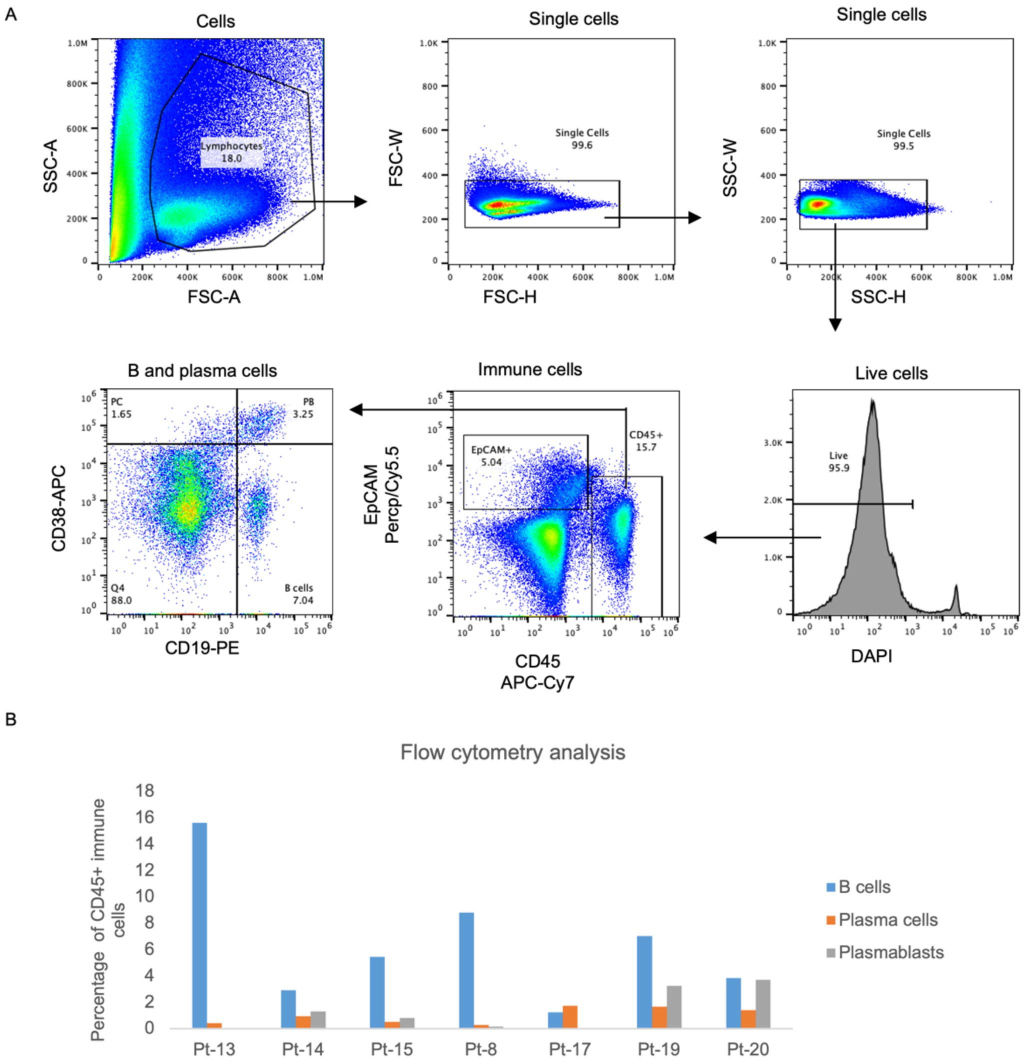

Figure S1. Characterization of B and plasma cells in primary PDAC microenvironment by flow cytometry for the samples used for single-cell sequencing.

(**A**) Representative flow cytometry gating strategy, shown by data from patient 19. Note that samples from patients 13, 14, 15 and 8, did not stain with EpCAM, thus the immune cells were gated with CD45$^+$ only. For samples 17, 19, and 20, EpCAM$^-$CD45$^+$ was used for gating of immune cells. (**B**) Percentage of B cells (CD19$^+$CD38$^{-/low}$), mature plasma cells (CD38^{hi}CD19$^-$, PC), and plasmablast cells (CD19$^+$CD38$^+$) of total CD45 immune cells in each of the seven samples used for single-cell sequencing.

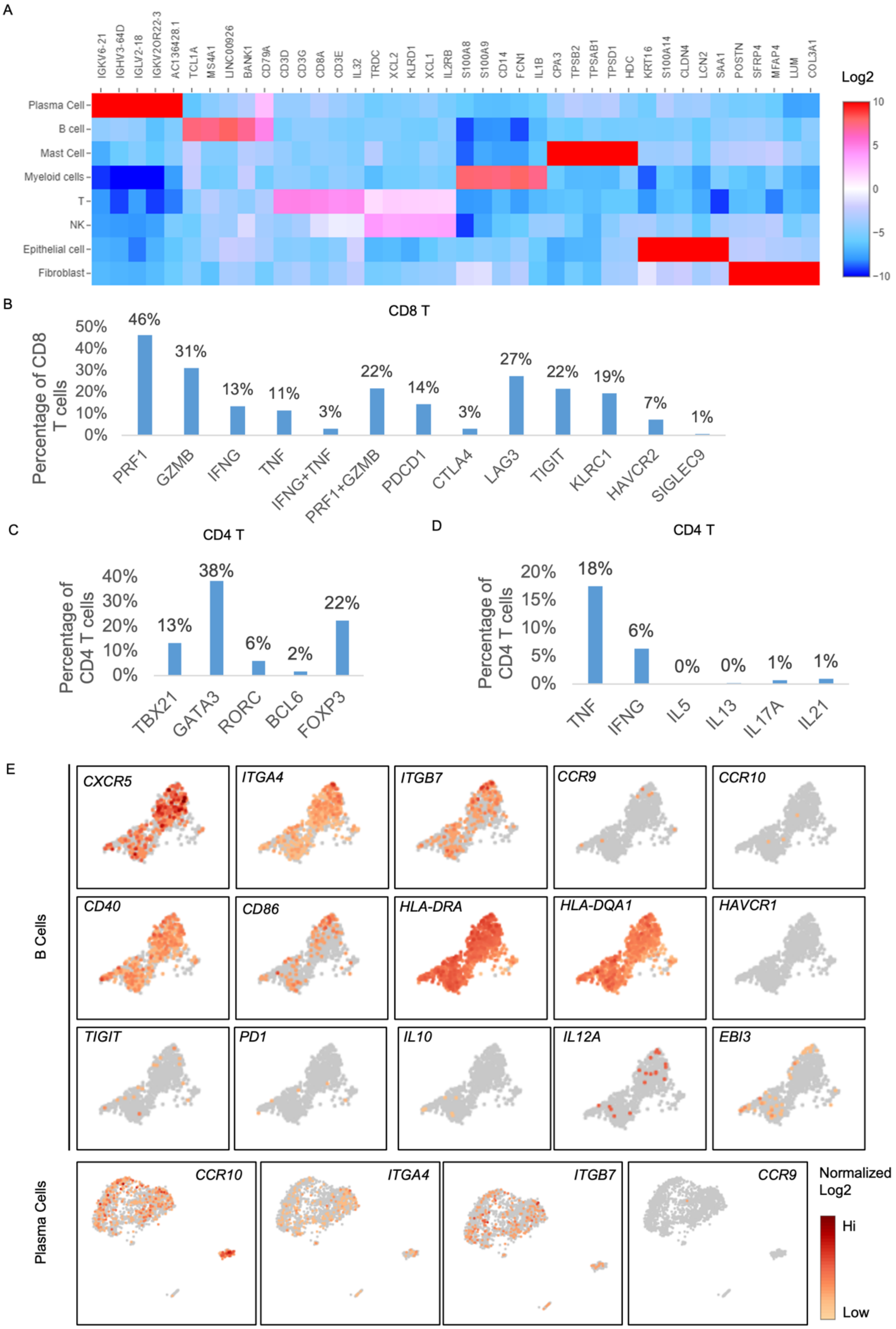
A
IGKV6-21
IGHV3-64D
IGLV2-18
IGKV2OR22-3
AC136428.1
TCL1A
MS4A1
LINC00926
BANK1
CD79A
CD3D
CD3G
CD8A
CD3E
IL32
TRDC
XCL2
KLRD1
XCL1
IL2RB
S100A8
S100A9
CD14
FCN1
IL1B
CPA3
TPSB2
TPSAB1
TPSD1
HDC
KRT16
S100A14
CLDN4
LCN2
SAA1
POSTN
SFRP4
MFAP4
LUM
COL3A1
Plasma Cell
B cell
Mast Cell
Myeloid cells
T
NK
Epithelial cell
Fibroblast
Log2
10
5
0
−5
−10
B
CD8 T
Percentage of CD8 T cells
50%
40%
30%
20%
10%
0%
46%
31%
13%
11%
3%
22%
14%
3%
27%
22%
19%
7%
1%
PRF1
GZMB
IFNG
TNF
IFNG+TNF
PRF1+GZMB
PDCD1
CTLA4
LAG3
TIGIT
KLRC1
HAVCR2
SIGLEC9
C
CD4 T
Percentage of CD4 T cells
40%
30%
20%
10%
0%
13%
38%
6%
2%
22%
TBX21
GATA3
RORC
BCL6
FOXP3
D
CD4 T
Percentage of CD4 T cells
20%
15%
10%
5%
0%
18%
6%
0%
0%
1%
1%
TNF
IFNG
IL5
IL13
IL17A
IL21
E
B Cells
CXCR5
ITGA4
ITGB7
CCR9
CCR10
CD40
CD86
HLA-DRA
HLA-DQA1
HAVCR1
TIGIT
PD1
IL10
IL12A
EBI3
Plasma Cells
CCR10
ITGA4
ITGB7
CCR9
Normalized Log2
Hi
Low

Figure S2. Characterization of primary PDAC immune cells microenvironment by scRNA-Seq. **(A)** Heatmap of the top five most upgraded genes in each cell cluster defined in Figure 1A. Color scale is log2 fold change. (**B-D**) The percentage of cells expressing selected gene markers in the clusters of CD8 T (**B**) and CD4 (**C**, **D**). (**E**) Selected genes expression in B cells and PCs.

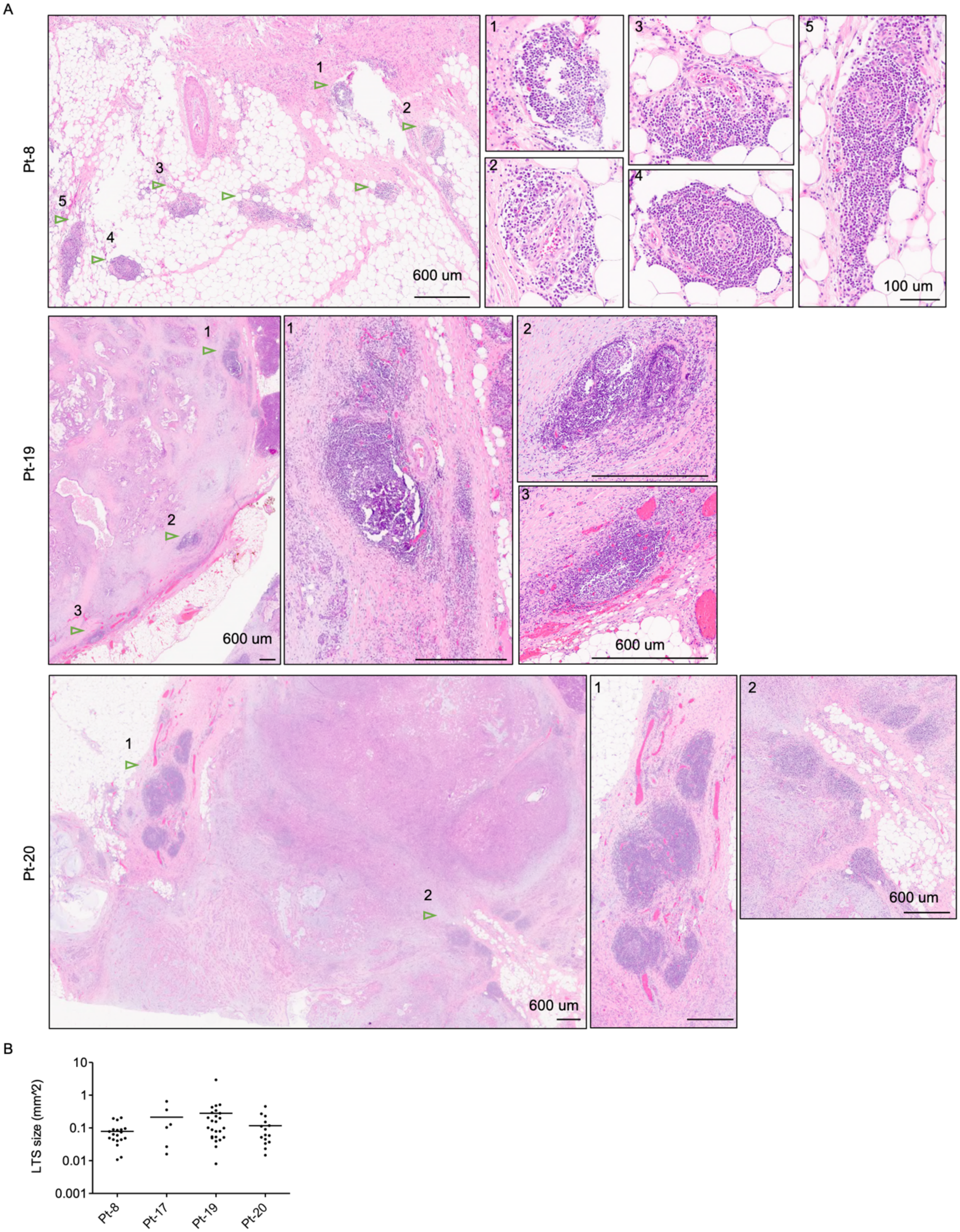
A
Pt-8
1
2
3
4
5
600 um
100 um
Pt-19
600 um
Pt-20
600 um
B
LTS size (mm^2)
10
1
0.1
0.01
0.001
Pt-8
Pt-17
Pt-19
Pt-20

Figure S3. Presence of TLS-like structures in PDAC.

(A) Examples of TLS-like structures in patients 8, 19 and 20 (TLS indicated by arrowhead and labeled by numbers). The zoom-in images were shown on the right. Scale bar is shown in figures. (**B**) Quantification and size distribution of the TLS in the four resected PDAC samples. Only TLS with size bigger than 0.01 mm^2 was analyzed. Individual data point and mean are shown in (**B**).

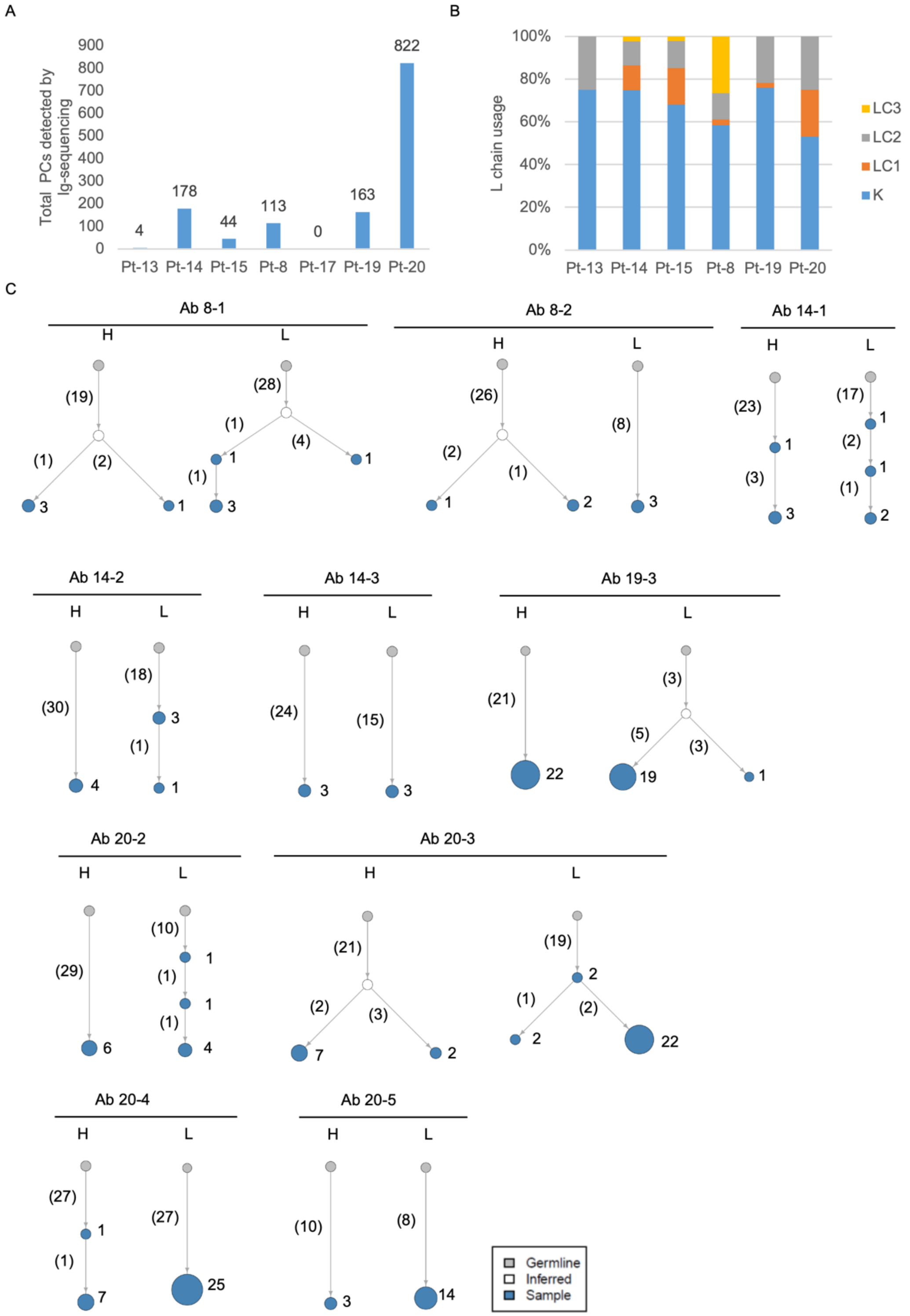
A
Total PCs detected by Ig-sequencing
900
800
700
600
500
400
300
200
100
0
4
178
44
113
0
163
822
Pt-13 Pt-14 Pt-15 Pt-8 Pt-17 Pt-19 Pt-20
B
L chain usage
100%
80%
60%
40%
20%
0%
Pt-13 Pt-14 Pt-15 Pt-8 Pt-19 Pt-20
LC3
LC2
LC1
K
C
Ab 8-1
Ab 8-2
Ab 14-1
Ab 14-2
Ab 14-3
Ab 19-3
Ab 20-2
Ab 20-3
Ab 20-4
Ab 20-5
H
L
Germline
Inferred
Sample

Figure S4. Ig sequencing summary and additional Ig sequences evolution trees from PCs in PDAC. **(A)** Total numbers of PCs detected by Ig sequencing, including PCs with paired H and L chains were sequenced (shown in Figure 2A) and PCs with only single H or L chains (unpaired) were sequenced. (**B**) Frequency of light constant chains usage among the PCs in individual samples. (**C**) Additional examples of antibody lineage evolution among top expanded PC clones screened in this study. Clone size is indicated by the size of the node (not scaled) and labeled by numbers on the right. The clone size includes both paired and unpaired chains. Number of somatic mutations in combined V(D)J regions is shown in parenthesis.

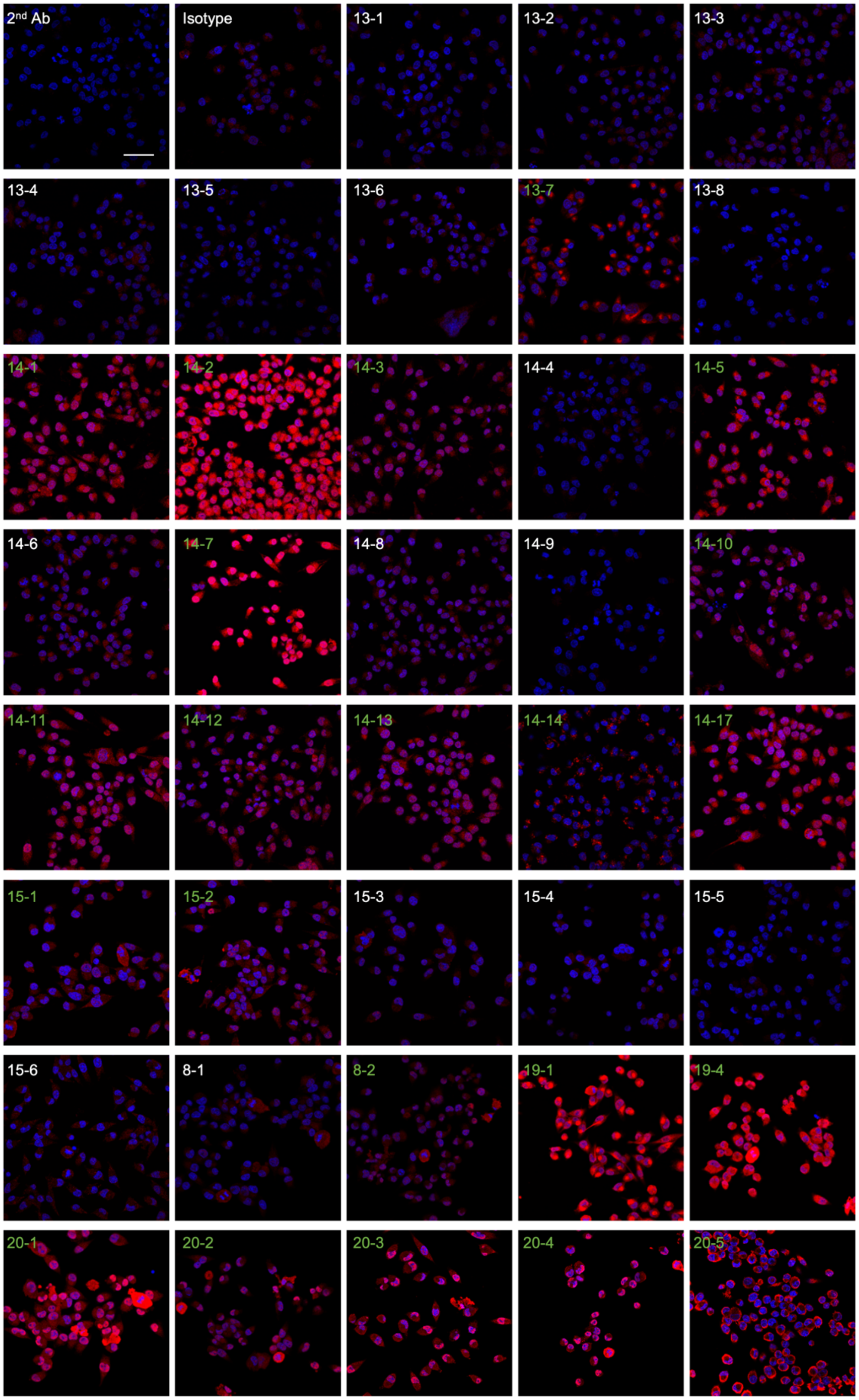
2nd Ab
Isotype
13-1
13-2
13-3
13-4
13-5
13-6
13-7
13-8
14-1
14-2
14-3
14-4
14-5
14-6
14-7
14-8
14-9
14-10
14-11
14-12
14-13
14-14
14-17
15-1
15-2
15-3
15-4
15-5
15-6
8-1
8-2
19-1
19-4
20-1
20-2
20-3
20-4
20-5

Figure S5. Antibodies staining atlas on MiaPaca2 cell line.

MiaPaca2 cells were stained with each antibody (red) and co-stained with DAPI (blue). Secondary antibody only (2nd Ab) and isotype IgG were used as control. Antibodies with positive staining are labeled green, and antibodies with negative staining are labeled white. Antibodies staining of 8-3, 19-3 and 15-7 were shown in the main figures and not included here. All images were acquired using the same confocal setting for isotype control staining, and the gain on the query antibody was reduced if the images were saturated. The experiment was repeated three times. Scale bar is 50um.

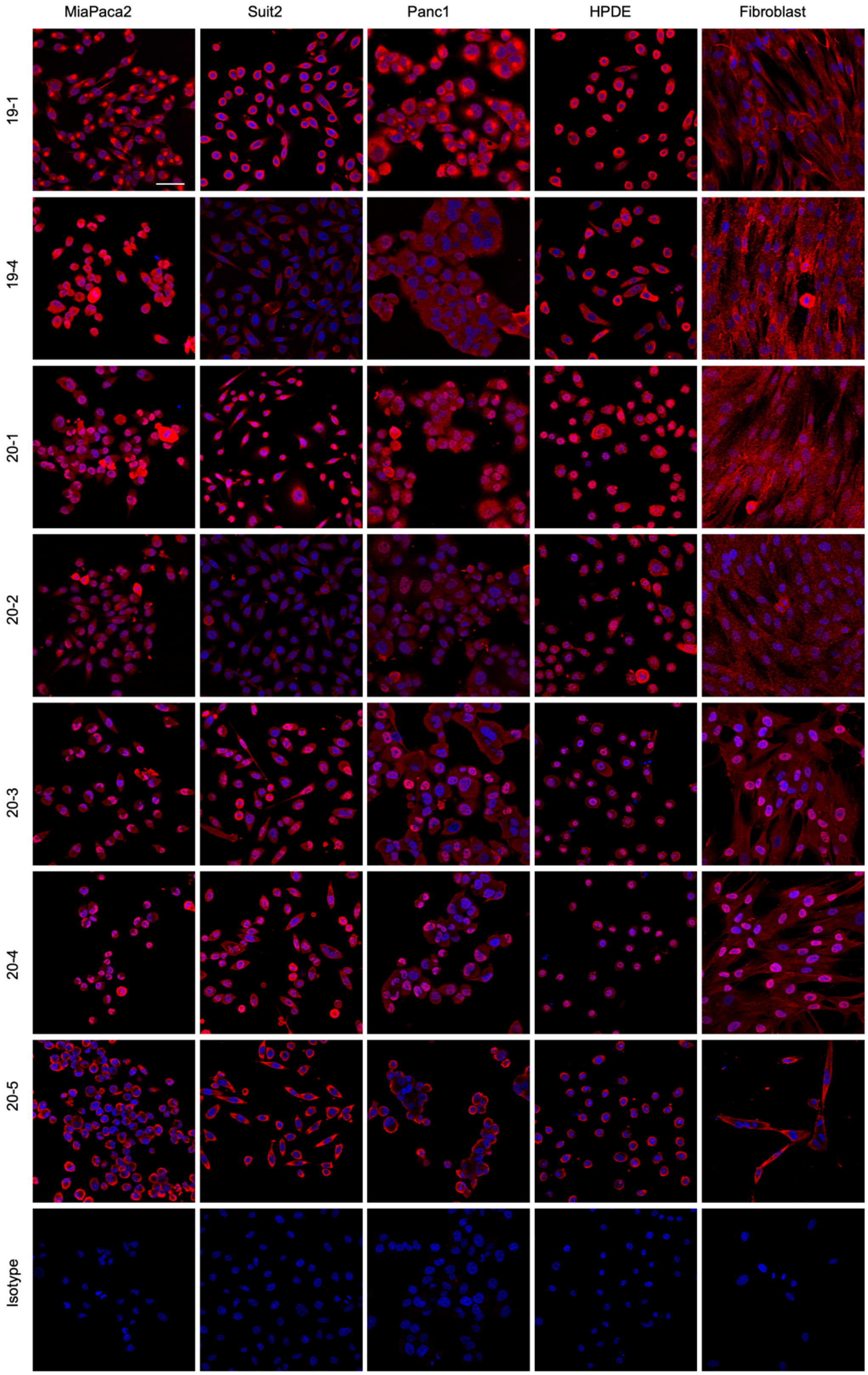
MiaPaca2
Suit2
Panc1
HPDE
Fibroblast
19-1
19-4
20-1
20-2
20-3
20-4
20-5
Isotype

Figure S6. Examples of antibodies staining in PDAC and non-cancer cell lines.

Cells were stained with each antibody (red) and co-stained with DAPI (blue). The experiment was repeated three times. Scale bar is 50um.

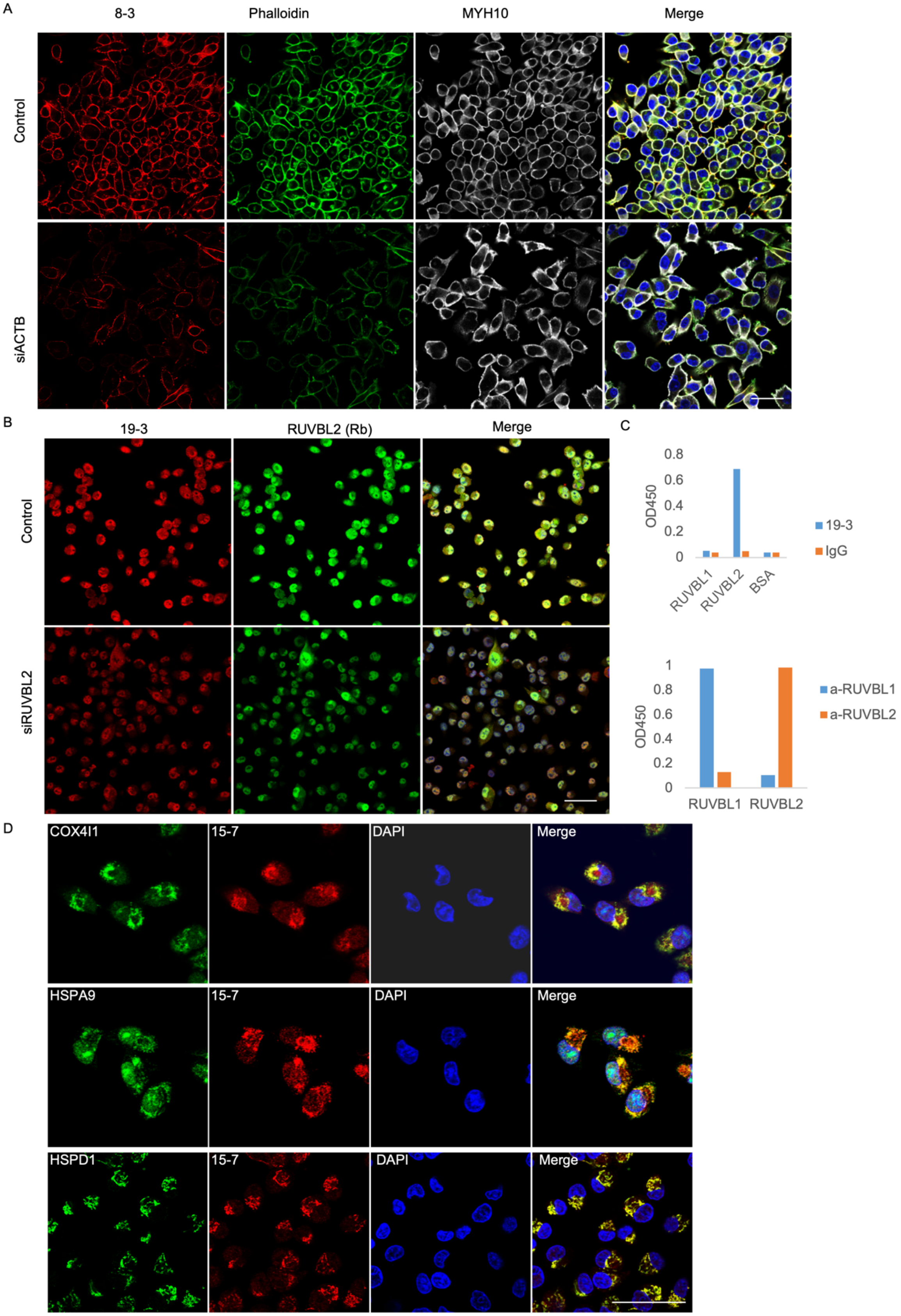

A
8-3
Phalloidin
MYH10
Merge
Control
siACTB
B
19-3
RUVBL2 (Rb)
Merge
Control
siRUVBL2
C
OD450
0.8
0.6
0.4
0.2
0
RUVBL1
RUVBL2
BSA
19-3
IgG
OD450
1
0.8
0.6
0.4
0.2
0
RUVBL1
RUVBL2
a-RUVBL1
a-RUVBL2
D
COX4I1
15-7
DAPI
Merge
HSPA9
15-7
DAPI
Merge
HSPD1
15-7
DAPI
Merge

Figure S7. Characterization of antibodies 8-3, 19-3 and 15-7.

(**A**) 8-3 staining after siRNA knockdown of *ACTB* in MiaPaca2 cells, co-stained with phalloidin, MYH10 antibody and DAPI. (**B**) Measurement of 19-3 binding to recombinant RUVBL1 and RUBL2 proteins by ELISA. Positive controls using polyclonal rabbit antibodies anti-RUVBL1 (a-RUVBL1) or anti-RUVBL2 (a-RUVBL2). (**C**) 19-3 staining after siRNA knockdown of *RUVBL2* in MiaPaca2 cells, co-stained with anti-RUVBL2 rabbit antibody and DAPI. (**D**) Co-staining of 15-7 with mitochondria markers COX4I1, HSPA9 and HSPD1 in MiaPaca2 cells. Each experiment was repeated twice. Scale bars in **A**, **C** and **D** are 50um.

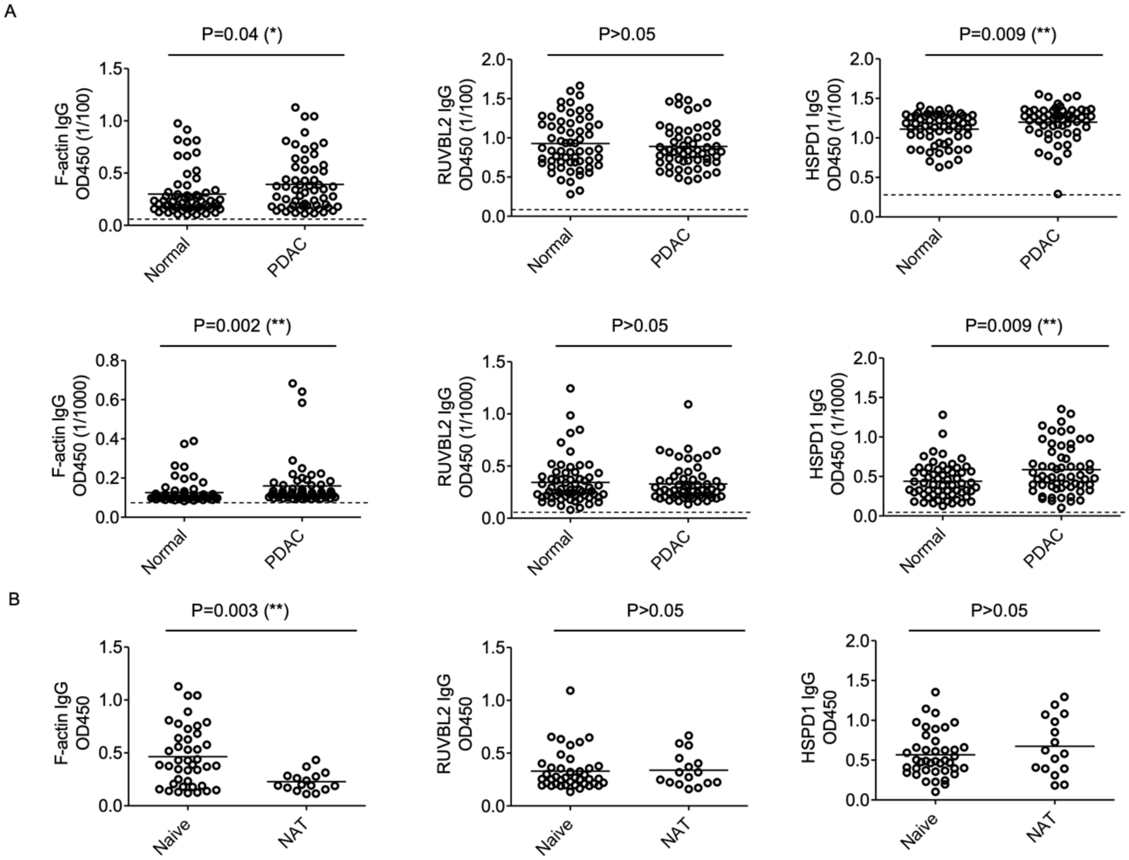

Figure S8. Comparison of plasma IgG titers to F-actin, RUVBL2 and HSPD1 between healthy donors and PDAC patients. (**A**) Comparison of IgG titer to F-actin, RUVBL2 and HSPD1 of plasma samples diluted at 1/100 or 1/1000 by ELISA. Background signals from secondary antibody only are shown in a dashed line. (**B**) Comparison of IgG response to F-actin, RUVBL2 and HSPD1 between naïve treated (naive) and neoadjuvant treated (NAT) PDAC patients. The non-parametric t-test is used for comparisons. The individual data points and mean are shown in all figures. The experiment was repeated twice.

Table S1. Human PDAC samples information for scRNA-Seq and Ig sequencing studies.

**Patient ID**	Pt-8	Pt-17	Pt-19	Pt-20	Pt-13	Pt-14	Pt-15
**Diagnosis**	PDAC	PDAC	PDAC	PDAC	PDAC	PDAC	PDAC
**Collection method**	resection	resection	resection	resection	FNA	FNA	FNA
**Age**	58	75	71	67	40	89	53
**Gender**	Male	Male	Female	Female	Female	Male	Female
**Race**	Caucasian	Caucasian	African-American	Asian	Latino/Hispanic	Caucasian	African American
**Neoadjuvant therapy**	No	No	No	No	No	No	No
**TNM stage**	pT2N2Mx	pT2N1Mx	pT2N1	pT2N0	NA	NA	NA
**Tumor size (cm)**	2.2*1.5*1	2.7*2.6*2.5	2.7*2.3*2.2	2.2*1.7*0.8	NA	NA	NA
**MSI status**	MSS	MSS	MSS	MSS	NA	NA	NA
**Tumor organoid available**	Yes	No	No	Yes	Yes	Yes	No
**Tumor section available**	Yes	Yes	Yes	Yes	No	No	No
**scRNA-Seq**	Yes	Yes	Yes	Yes	Yes	Yes	Failed
**Ig sequencing**	Yes	Yes (low B and PC cells)	Yes	Yes	Yes	Yes	Yes

Abbreviations: FNA, fine needle aspiration; MSI, microsatellite instable; MSS, microsatellite stable; NA, not available/appliable

Table S3. Human samples information for plasma studies.

		Normal (n=61)		PDAC (n=59)		Comparison
		No.	Ratio	No.	Ratio	p value
Gender	Male	22	36%	22	37%	n.s.
	Female	39	64%	32	54%	
	Unknown	0	0%	5	8%	
Age	Median (range)	36	17-66	69.5	49-89	<0.0001
	Unknown	0		5		
Race	Asian	7	11%	5	8%	<0.001
	African American	13	21%	7	12%	
	Caucasian	8	13%	40	68%	
	Hispanic	33	54%	2	3%	
	NA	0	0%	5	8%	
Treatment	Neoadjuvant	-		16	27%	
	No pre-treatment	-		41	69%	
	NA	-		2	3%	
T stage	T1	-		4	7%	
	T2	-		26	44%	
	T3	-		13	22%	
	T4	-		1	2%	
	NA	-		15	25%	
N stage	N0	-		15	25%	
	N1	-		17	29%	
	N2	-		12	20%	
	NA	-		15	25%	
M stage	Mx	-		54	92%	
	M1	-		5	8%	
